# Loss of *Dnmt3a* impairs hematopoietic homeostasis and myeloid cell skewing via the PI3Kinase pathway

**DOI:** 10.1172/jci.insight.163864

**Published:** 2023-05-08

**Authors:** Lakshmi Reddy Palam, Baskar Ramdas, Katelyn Pickerell, Santhosh Kumar Pasupuleti, Rahul Kanumuri, Annamaria Cesarano, Megan Szymanski, Bryce Selman, Utpal P. Dave, George Sandusky, Fabiana Perna, Sophie Paczesny, Reuben Kapur

**Affiliations:** 1 Department of Pediatrics, Herman B Wells Center for Pediatric Research,; 2 Department of Medicine,; 3 Department of Pathology and Laboratory Medicine, and; 4 Division of Hematology/Oncology, Department of Medicine, Indiana University School of Medicine, Indianapolis, Indiana, USA.; 5 Department of Microbiology and Immunology, Medical University of South Carolina, Charlestown, South Carolina, USA.

**Keywords:** Hematology, Hematopoietic stem cells, Leukemias

## Abstract

Loss-of-function mutations in the *DNA methyltransferase 3A (DNMT3A*) are seen in a large number of patients with acute myeloid leukemia (AML) with normal cytogenetics and are frequently associated with poor prognosis. *DNMT3A* mutations are an early preleukemic event, which — when combined with other genetic lesions — result in full-blown leukemia. Here, we show that loss of *Dnmt3a* in hematopoietic stem and progenitor cells (HSC/Ps) results in myeloproliferation, which is associated with hyperactivation of the phosphatidylinositol 3-kinase (PI3K) pathway. PI3Kα/β or the PI3Kα/δ inhibitor treatment partially corrects myeloproliferation, although the partial rescue is more efficient in response to the PI3Kα/β inhibitor treatment. In vivo RNA-Seq analysis on drug-treated *Dnmt3a^–/–^* HSC/Ps showed a reduction in the expression of genes associated with chemokines, inflammation, cell attachment, and extracellular matrix compared with controls. Remarkably, drug-treated leukemic mice showed a reversal in the enhanced fetal liver HSC-like gene signature observed in vehicle-treated *Dnmt3a^–/–^* LSK cells as well as a reduction in the expression of genes involved in regulating actin cytoskeleton-based functions, including the RHO/RAC GTPases. In a human PDX model bearing *DNMT3A* mutant AML, PI3Kα/β inhibitor treatment prolonged their survival and rescued the leukemic burden. Our results identify a potentially new target for treating *DNMT3A* mutation–driven myeloid malignancies.

## Graphical abstract

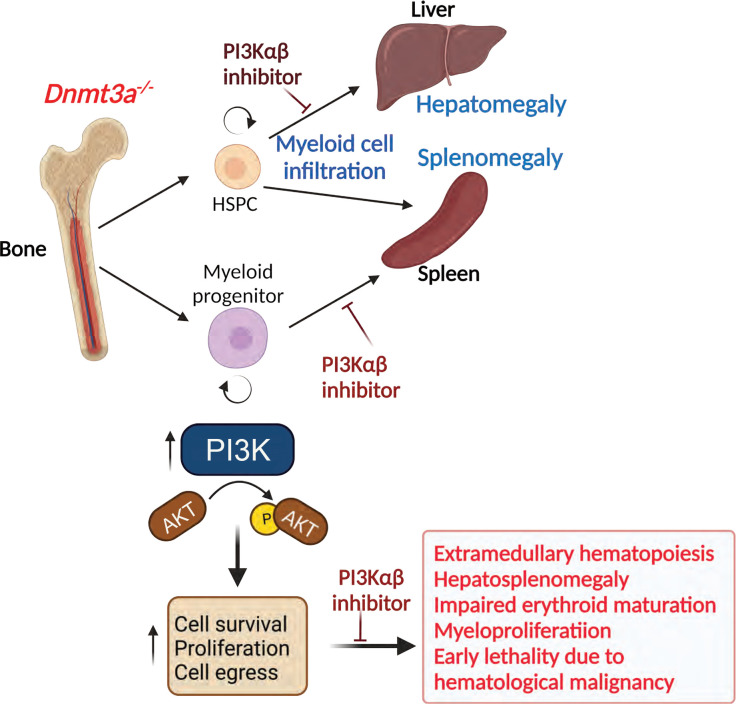

## Introduction

*DNA methyl transferase 3A* (*DNMT3A*) is one of the most frequently mutated genes in patients with myeloid malignancies. *DNMT3A* mutations are found in approximately 25% of patients with acute myeloid leukemia (AML) and close to 10% in patients with myeloproliferative neoplasms (MPN) and myelodysplastic syndromes (MDS) (1–4). Bulk sequencing studies on the peripheral blood (PB) of normal individuals with no overt signs of myeloid malignancies have revealed that *DNMT3A* mutations occur as preleukemic somatic mutations that can be later detected in patients with full-blown AML (5–8). Somatic *DNMT3A* mutations are highly associated with age-related clonal hematopoiesis (9–11). In this regard, *DNMT3A* R882H mutation, which is frequently seen in humans, functionally acts in a dominant negative manner, leading to aberrant hypomethylation, similar to that observed in *Dnmt3a^–/–^* cells (12). In mice, loss of *Dnmt3a* results in enhanced hematopoietic stem cell self-renewal, proliferation, and myeloid cell bias (13). Additionally, *Dnmt3a* deletion in hematopoietic stem cells induces leukemic transformation with accumulation of additional mutations in genes involved in regulating stem cell growth and survival, including *cKit*, *Flt3*, and *Ras* (14, 15). Although patients with *DNMT3A* mutations show hepatosplenomegaly and lymphadenopathy due to extramedullary chronic myelomonocytic leukemia (CMML) (13) compared with patients with WT *DNMT3A*; the signaling pathways that induce such profound hematopoietic dysregulation are poorly understood. Consistent with these observations, loss of *Dnmt3a* in mice results in reduced overall survival as well as hepatosplenomegaly, which is associated with myeloid cell infiltration in various tissues, myeloproliferation, and liver-specific expansion of hematopoietic stem and progenitor cells (HSC/Ps) (13). Intriguingly, hyperactivated phosphatidylinositol 3-kinase (PI3K) signaling due to specific loss of *Pten* in HSCs also results in HSC expansion, mobilization, hepatosplenomegaly, and extramedullary hematopoiesis, similar to *Dnmt3a^–/–^* mice (16, 17). Several reports point to hyperactivation of PI3K signaling in AML, and activated AKT is commonly associated with poor overall survival of patients with AML as well as with a less complete response (CR) rate to chemotherapy (18–23). However, little is known about the signaling pathways that are modulated as a result of *Dnmt3a* mutations or its loss of function in HSC/Ps.

Here, we sought to determine the mechanisms by which loss of *Dnmt3a* induces hematopoietic dysregulation in *Dnmt3a^–/–^* mice. We show that loss of Dnmt3a in HSC/Ps results in hypersensitivity to hematopoietic cytokines, which is associated with increased expression of genes in the PI3K pathway, including a decrease in the expression of *Pten*. Loss of the regulatory components of the PI3K signaling cascade in the setting of *Dnmt3a^–/–^* HSC/Ps partially restores cytokine-induced hypersensitivity. In a transplant setting, treatment of mice bearing *Dnmt3a^–/–^* cells with a PI3Kα/β or PI3Kα/δ inhibitor rescues *Dnmt3a* mutant–associated hematologic abnormalities, including hepatosplenomegaly, myeloid cell infiltration in the tissues, and anemia as well as thrombocytopenia. This restoration is associated with an in vivo suppression in the activation of the PI3K/AKT pathway in drug-treated LSK cells from *Dnmt3a^–/–^* mice. While the PI3K pathway has been targeted for treatment in some lymphoid malignancies, its role in DNMT3a loss-of-function–associated development of myeloid malignancies has not been investigated.

## Results

**Figure 1:**
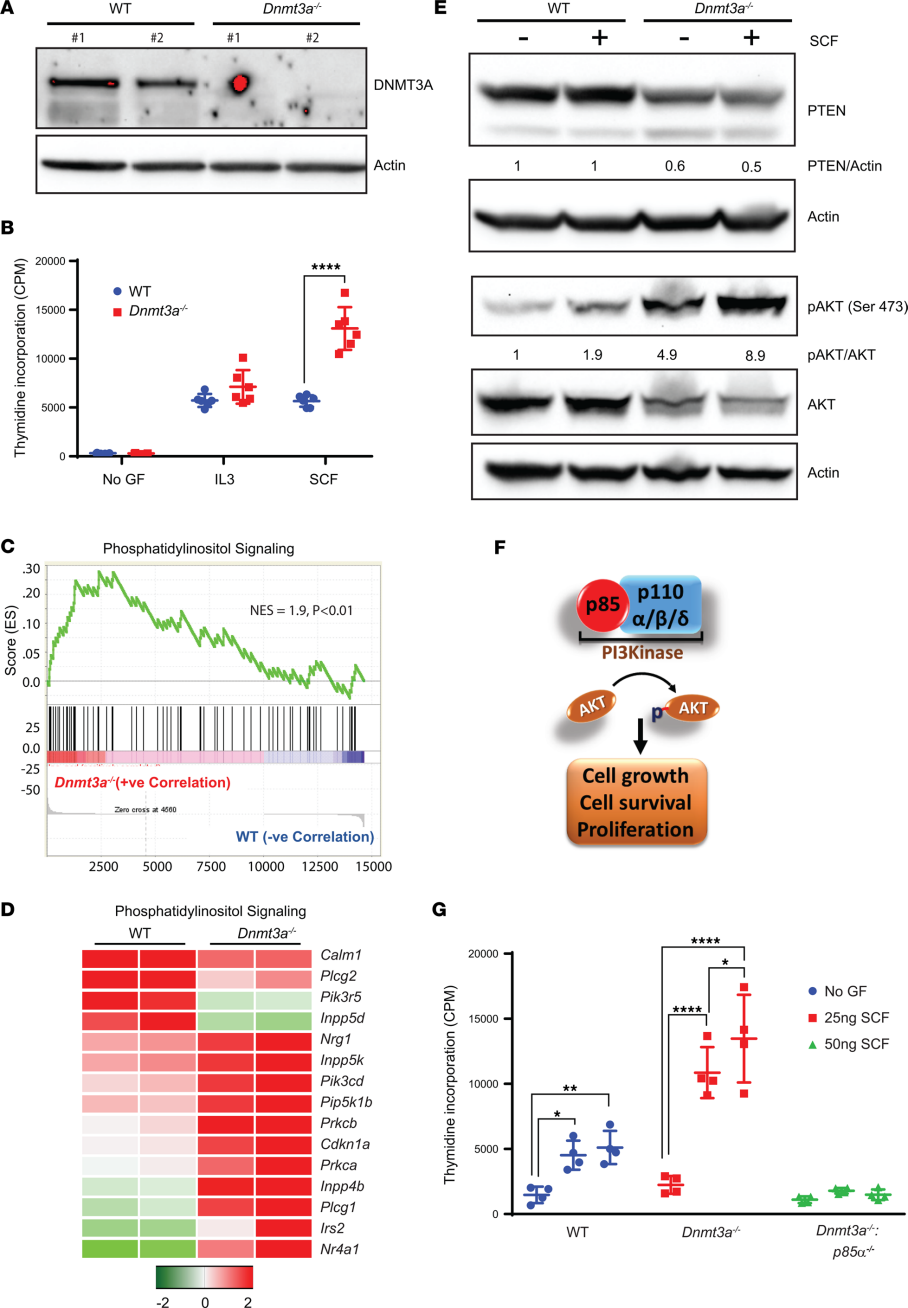
Loss of *Dnmt3a* in BMNC results in increased cell proliferation via the PI3K pathway. (**A**) WT and *Dnmt3a^fl/fl^:Mx-Cre* mice were administered poly I:C at 12–16 weeks of age, and BMNCs were subjected to Western blot analysis to detect the presence of DNMT3a protein. Two independent WT and *Dnmt3a^–/–^* mouse–derived BMNCs were utilized for these experiments. (**B**) BMNCs from WT or *Dnmt3a^–/–^* mice were cultured in media supplemented with SCF (50 ng/mL) or IL-3 (10 ng/mL) or no growth factor for 48 hours. Cell proliferation was evaluated by [^3^H] thymidine incorporation. Counts per minute (CPM) are shown. Three independent experiments, *n* = 6, mean ± SD, ****P < 0.00005. BM cells collected from WT or *Dnmt3a^–/–^* mice were subjected to RNA isolation and, subsequently, next-generation sequencing. (**C** and **D**) GSEA revealed an enrichment for genes in the PI3K signaling pathway (**C**), and upregulation of specific genes in the PI3K pathway are shown in the heatmap (**D**). (**E**) BMNCs from WT or *Dnmt3a^–/–^* mice were starved of growth factors and stimulated with SCF, followed by Western blot analysis. (**F**) Class I PI3K complex is composed of p85 regulatory subunit and p110α, p110β, and p110δ catalytic subunits. Activated PI3K signaling regulates AKT phosphorylation, which in turn promotes cell grow, cell survival, and proliferation. (**G**) WT, *Dnmt3a^fl/fl^:Mx-Cre*, and *Dnmt3a^fl/fl^*:*p85*α*^fl/fl^*:*Mx-Cre* mice were administered poly I:C at 12–16 weeks of age. BMNCs were collected from 3 different mice of each genotype and stimulated with SCF (25 ng/mL or 50 ng/mL) or in the absence of growth factors for 48 hours. Cell proliferation was evaluated by [^3^H] thymidine incorporation. Counts per minute (CPM) are shown. Experiment performed 3 times, and representative experiment shown with *n* = 4, mean ± SD, *P < 0.05, **P < 0.005, ****P < 0.00005. Two-way ANOVA, Tukey’s multiple-comparison test (**B** and **C**).

For studies performed here, we used the *Mx1-Cre* inducible conditional *Dnmt3a*-KO mouse model, which has been previously described and mimics a significant number of cardinal features seen in mice bearing the R882 mutation, which is considered one of the most common *DNMT3A* mutations in humans (24, 25). We first evaluated the impact of *Dnmt3a* loss in myeloid lineage–derived cells from the BM mononuclear cells (BMNC) of *Dnmt3a^–/–^* mice, cultured in the presence of IL-3. As seen in Figure 1A, Western blot analysis on cells derived from 2 independent *Dnmt3a*-null mice showed loss of DNMT3a protein compared with WT controls. To assess if loss of *Dnmt3a* modulates the growth potential of myeloid cells, we subjected these cells to an in vitro proliferation (thymidine incorporation) assay to assess their response to cytokines such as stem cell factor (SCF) and IL-3. As seen in Figure 1B, an increase in the proliferation of *Dnmt3a^–/–^* BMNCs was observed in the presence of SCF compared with controls. To assess the mechanisms by which loss of *Dnmt3a* in BMNCs might induce hyperproliferation of these cells in response to cytokines such as SCF, we performed RNA-Seq analysis, followed by gene set enrichment analysis (GSEA) on BMNCs from WT and *Dnmt3a^–/–^* mice. As seen in Figure 1C, GSEA demonstrated an enrichment and upregulation in the phosphatidylinositol (PI3K) signaling pathway in *Dnmt3a^–/–^* BMNCs compared with controls. Figure 1D shows a heatmap depicting some of the upregulated genes within the PI3K pathway in *Dnmt3a^–/–^* cells as opposed to WT cells. An increase in the transcript levels of PI3K signaling components, such as *Pik3cd* (Phosphatidylinositol-4,5-Bisphosphate 3-Kinase Catalytic Subunit δ), *Pip5k1b* (Phosphatidylinositol-4-Phosphate 5-Kinase Type 1 β), and *INPP4B* (Inositol Polyphosphate-4-Phosphatase Type II B), was observed in *Dnmt3a^–/–^* cells. An increase in the transcript levels of PI3K signaling–associated components, such as *Prkcb*: (Protein Kinase C β), *Pkca* (Protein kinase C α), *Plcg1* (Phospholipase C Ɣ 1), *Irs2* (Insulin Receptor Substrate 2), and *Nr4a1* (Nuclear Receptor Subfamily 4 Group A Member 1), was observed in *Dnmt3a^–/–^* cells relative to controls. PI3K δ is expressed specifically in leukocytes and plays a major role in cell proliferation/migration (26). *Pip5k1b* plays a role in cellular processes such as vesicle trafficking, cell adhesion, and motility. *Inpp4b* plays a role in late stages of micropinocytosis by acting on PI-3,4-bisphosphate in membrane ruffles. PKCα and -β kinases modulate cellular processes downstream from PI3K signaling. All of these key components of the PI3K signaling cascade were upregulated in *Dnmt3a^–/–^* cells as opposed to WT cells (Figure 1D).

Given these transcriptomic changes, we next performed biochemical studies to assess if we could detect any perturbation in the PI3K signaling pathway at the biochemical level. As seen in Figure 1E, an increase in the activation of AKT (Ser 473) was observed in *Dnmt3a^–/–^* BMNCs stimulated with SCF compared with controls. Baseline activation of AKT was also enhanced in *Dnmt3a^–/–^* cells compared with controls. The increase in AKT activation in *Dnmt3a^–/–^* cells was associated with a reduction in the relative levels of PTEN, a lipid phosphatase and a negative regulator of the PI3K signaling pathway (Figure 1E). We next assessed the extent to which the PI3K/AKT pathway contributes to the enhanced proliferation noted in *Dnmt3a^–/–^* cells. PI3K is a multiprotein complex consisting of a p85 regulatory subunit and one of the 3 p110 (α, β, or δ) catalytic subunits (Figure 1F). p85 regulatory subunit plays a critical role in regulating the stability of all 3 catalytic subunits (27). To directly assess the role of PI3K in DNMT3a loss–induced cytokine hyperproliferation in BMNCs, we performed a genetic intercross between *Dnmt3a^–/–^* mice and *p85*α*^–/–^* mice to generate mice deficient in the expression of both *Dnmt3a* and *p85*α. BMNCs from these mice and controls were subjected to a proliferation assay in the presence of SCF. As seen in Figure 1G, a reduction in SCF-induced hyperproliferation was observed in cells derived from mice deficient in the expression of both *Dnmt3a* and *p85α* compared with controls. These data suggest that loss of *Dnmt3a* induces hyperactivation of the PI3K pathway, inhibition of which substantially restores the hyperproliferation seen in these cells in response to cytokines such as SCF.

**Figure 2:**
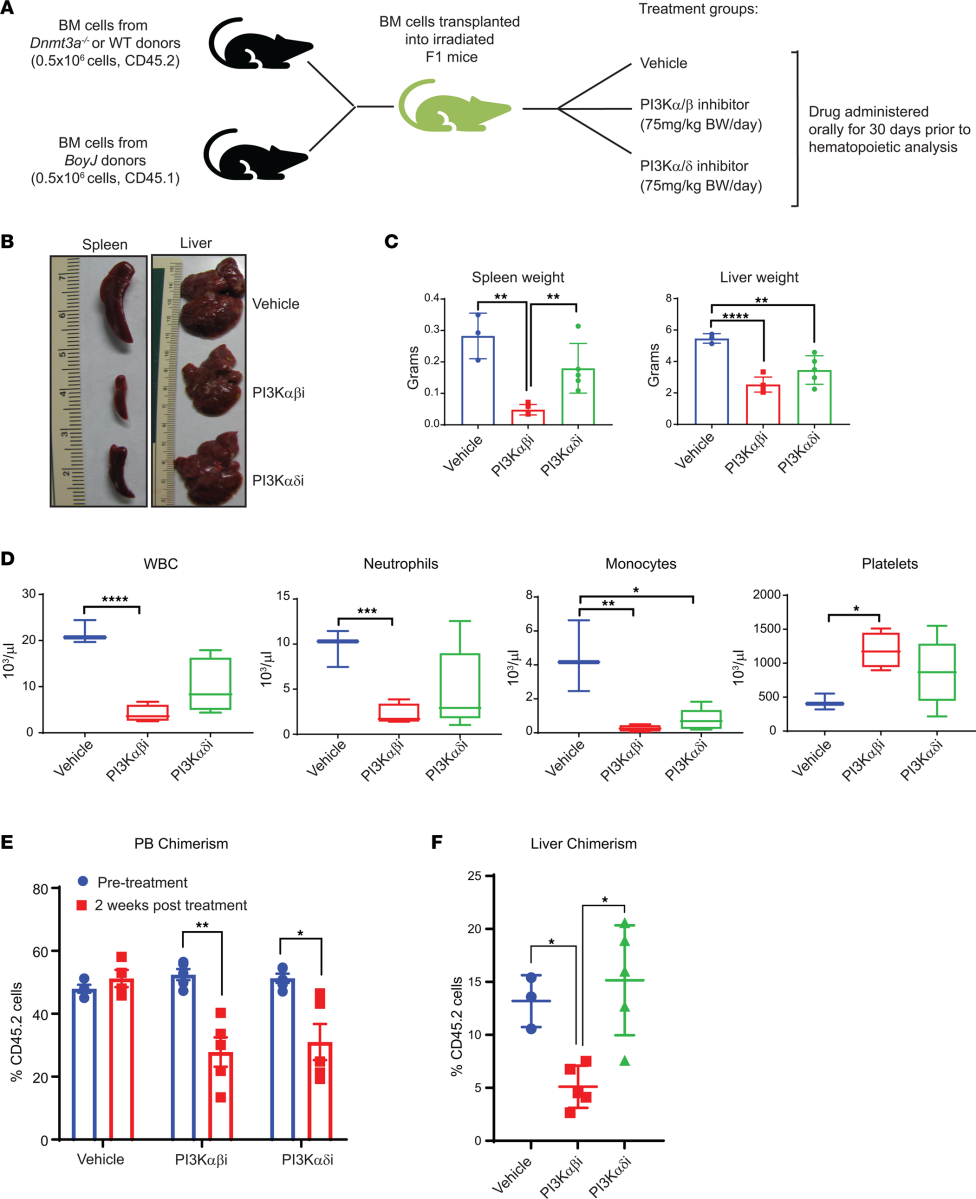
PI3K αβ inhibition modulates *Dnmt3a* loss–induced myeloid leukemia development. (**A**) Donor cells from *Dnmt3a^–/–^* mice were mixed with BoyJ cells in 1:1 ratio (0.5 × 10^6^ versus 0.5 × 10^6^) for a competitive transplantation assay. Six weeks after transplantation, mice were treated with vehicle or the PI3K αβ inhibitor (Bay1082439; 7 mg/kg body weight) or PI3K αδ inhibitor (GDC-0941; 75 mg/kg body weight) for 30 days, and mice were analyzed. (**B**) Representative images of liver and spleen from vehicle- and drug-treated mice are shown. (**C**) Quantitative assessment of spleen and liver weights from the indicated groups. *n* = 3–5, mean ± SD, **P < 0.005, ****P < 0.00005. (**D**) PB counts from mice described in **B** and **C** before they were sacrificed. *n* = 3–5, *P < 0.05, **P < 0.005, ***P < 0.0005, ****P < 0.00005. The boxes shown with lower and upper quartiles separated by the median (horizontal line), and the whiskers extend to the minimum and maximum values. (**E** and **F**) Mice described in **A** were analyzed for PB chimerism 2 weeks after drug treatment and after 30 days after drug treatment for liver chimerism. Chimerism was assessed by staining the cells using an anti-CD45.2 antibody and flow cytometry. *n* = 3–5, mean ± SEM, *P < 0.05, **P < 0.005. One-way ANOVA in **C**, **D**, and **F**; 2-way ANOVA in **E** with Tukey’s multiple comparison test performed.

Given these observations, we next determined if inhibition of PI3K signaling in mice transplanted with *Dnmt3a^–/–^* HSC/Ps rescues their growth. To test this, we transplanted 0.5 million *Dnmt3a^–/–^* whole BM cells and 0.5 million *BoyJ* cells into lethally irradiated F1 mice (Figure 2A). Six weeks after transplantation, once stable engraftment was achieved, mice were treated with either a PI3K α/β inhibitor or a α/δ inhibitor (Figure 2A). Bay1082439 is a PI3K α/β–specific inhibitor and is in clinical trials for treatment of advanced solid tumors (NCT01728311). GDC-0941 is a well-known PI3K α/δ inhibitor also in clinical development for treating solid tumors (28). Thirty days after drug treatment, mice were sacrificed, and tissues were harvested for further analysis. Figure 2, B and C, shows significant hepatosplenomegaly, normally observed in malignant *Dnmt3a^–/–^* mice, which is partially restored in *Dnmt3a^–/–^* mice treated with either of the 2 PI3K inhibitors; however, the restoration was more dramatic in *Dnmt3a^–/–^* mice treated with Bay1082439, a PI3K α/β–specific inhibitor, compared with GDC-0941, a PI3K α/δ inhibitor (Figure 2, C and D). PB analysis of drug-treated mice showed a correction in total white blood cell counts, neutrophil, and monocyte counts as well as correction in thrombocytopenia associated with these mice (Figure 2D). Consistent with the reduction in spleen size and PB counts in drug-treated mice, overall engraftment of *Dnmt3a^–/–^* cells was reduced in PI3K inhibitor–treated mice as early as 2 weeks after drug treatment (Figure 2E). In the liver, the frequency of *Dnmt3a^–/–^* cells was reduced in the presence of the PI3K α/β inhibitor compared with the α/δ inhibitor (Figure 2F). Taken together, these results suggest that targeting the PI3K signaling pathway in *Dnmt3a* mutant–bearing cells may play a role in inhibiting disease pathogenesis and suggest that certain catalytic subunits of PI3K may be differentially utilized by these cells to regulate leukemic cell growth.

**Figure 3:**
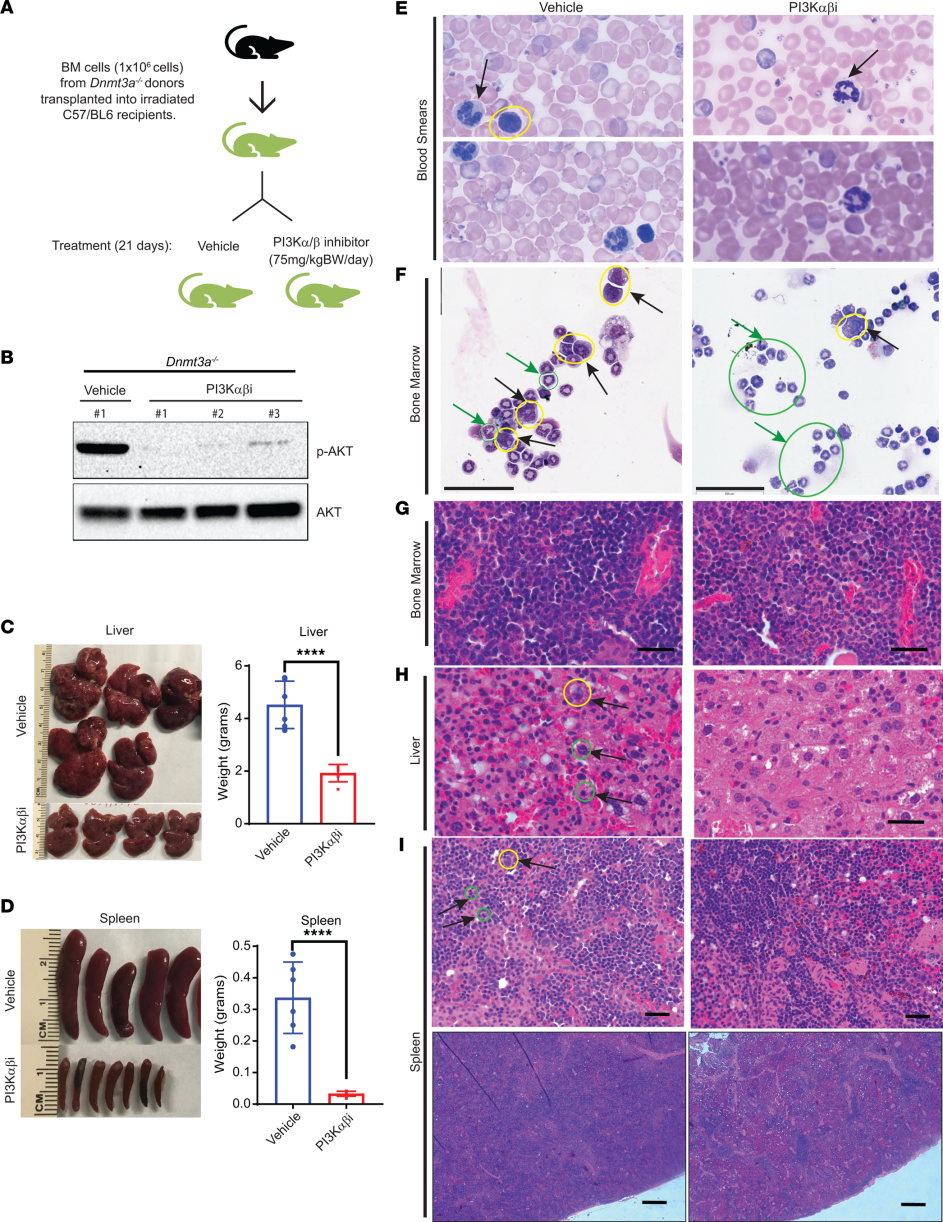
PI3K αβ inhibition rescues tissue myeloid cell infiltration as well as hepatosplenomegaly due to loss of *Dnmt3a* in HSC/Ps. (**A**) BM cells from *Dnmt3a^–/–^* mice were transplanted into lethally irradiated C57BL/6 mice, and 6 weeks after transplantation, mice were treated with vehicle or the PI3K αβ inhibitor (Bay1082439; 75 mg/kg body weight) for 21 days. (**B**) BM bearing *Dnmt3a^–/–^* cells from mice treated with vehicle or the PI3K αβ inhibitor were subjected to Western blot analysis to analyze pAKT and total AKT protein levels. Numbers at the top represent the number of mice used for these experiments. (**C** and **D**) Images of spleens and livers from vehicle- and drug-treated mice and respective quantitative assessment of liver and spleen weights from vehicle- or drug-treated mice. *n* = 5–6, mean ± SEM, unpaired *t* test (2-tailed), ****P < 0.00005. (**E** and **F**) Peripheral blood smears and BM cytospin preparations showing reduced dysplasia of myeloid and erythroid lineage cells in PI3K αβ inhibitor–treated mice compared with vehicle group. Arrows in blood smears indicate segmented neutrophils. Yellow circle indicates myeloblasts, and green circles/green arrows indicate metamyelocytes. PB smear images shown at 100***×*** magnification (**E**). Scale bar: 60 μm (**F**). (**G**) Representative images for H&E-stained sections of BM. Scale bar: 50μm (**H**) H&E-stained liver sections showing increased sinusoidal spaces with reduced immature myeloid cell infiltration in in PI3Kαβ inhibitor treatment condition compared with vehicle treatment. Immature myeloid precursor Metamyelocytes (yellow circle) and proliferating myeloid cells (green circles) in vehicle-treated mice are indicated. Scale bar: 50 μm (**I**) H&E-stained spleen sections showing reduced immature myeloid cell infiltration in the PI3Kαβ inhibitor treatment condition compared with vehicle treatment. Yellow circle indicates dysplastic megakaryocyte. More proliferating myeloid cells in vehicle-treated mice (green circles indicate mitotic figures of myeloproliferation). Scale bar: 50 μm (upper panel), 1,000 μm (lower panel).

Given the stronger impact of the PI3K α/β–specific inhibitor in reducing the burden of hepatosplenomegaly, we analyzed the impact of this inhibitor in more detail in lethally irradiated mice transplanted with only the *Dnmt3a^–/–^* BM cells in the absence of any WT competitor cells. Six weeks after transplantation, once stable engraftment was achieved, recipient mice were treated with the drug for 21 days, and tissues were collected for analysis (Figure 3A). Western blot analysis showed a decrease in AKT (Ser473) activation in *Dnmt3a^–/–^* cells treated with the PI3K α/β–specific inhibitor (Figure 3B). Consistent with our results utilizing a competitive BM transplant strategy (Figure 2), treatment of mice bearing whole BM cells from *Dnmt3a^–/–^* mice with the PI3K α/β–specific inhibitor also resulted in reduced liver size/weight as well as spleen size and weight (Figure 3, C and D). PB smears and BM cytospins showed a correction in erythroid cell hyperplasia (Figure 3, E and F).

**Figure 4:**
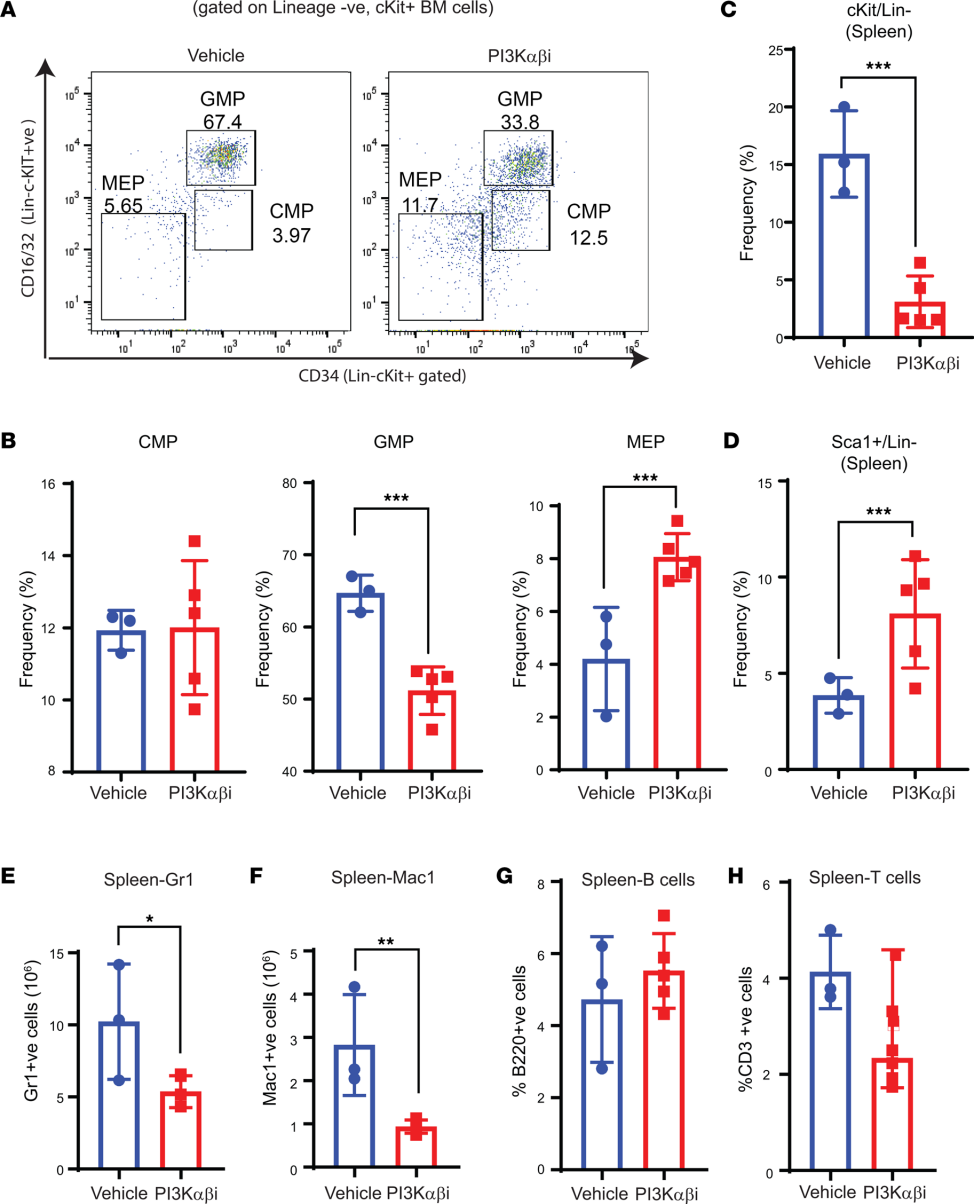
PI3K αβ inhibition decreases myeloid progenitors and improves megakaryocyte-erythrocyte progenitors in *Dnmt3a^–/–^* malignant mice. (**A** and **B**) Flow cytometry analysis was performed on BM cells from mice transplanted with *Dnmt3a^–/–^* cells and treated with the PI3K αβ inhibitor. Representative dot plots and quantitative data showing the frequency of Lin^–^/c-KIT^+^ myeloid progenitors: CMPs, GMPs, and MEPs. *n* = 3–5, mean ± SEM, ***P = 0.0005. (**C** and **D**) Spleen cells collected from vehicle- or PI3Kαβ inhibitor–treated mice as in **A** were subjected to flow cytometry analysis to detect Lin^–^c-KIT^+^Sca-1^+^ cells. Quantitative data showing reduced c-KIT^+^ (Lin^–^) myeloid progenitor cells (**C**) and increased differentiated c-KIT^–^ spleen cells (**D**) in PI3K αβ inhibitor–treated group compared with controls. *n* = 3–5, mean ± SEM, ***P = 0.0005. (**E**–**H**) Flow cytometry was performed on spleen cells from vehicle or PI3K αβ inhibitor–treated mice as in **A**. Quantitative data showing a reduction in myeloid cell burden in the spleen from drug treated *Dnmt3a^–/–^* mice compared with controls. *n* = 3–5, mean ± SEM, *P = 0.05, **P = 0.005, unpaired *t* test (2-tailed) performed (**B**–**H**).

**Figure 5:**
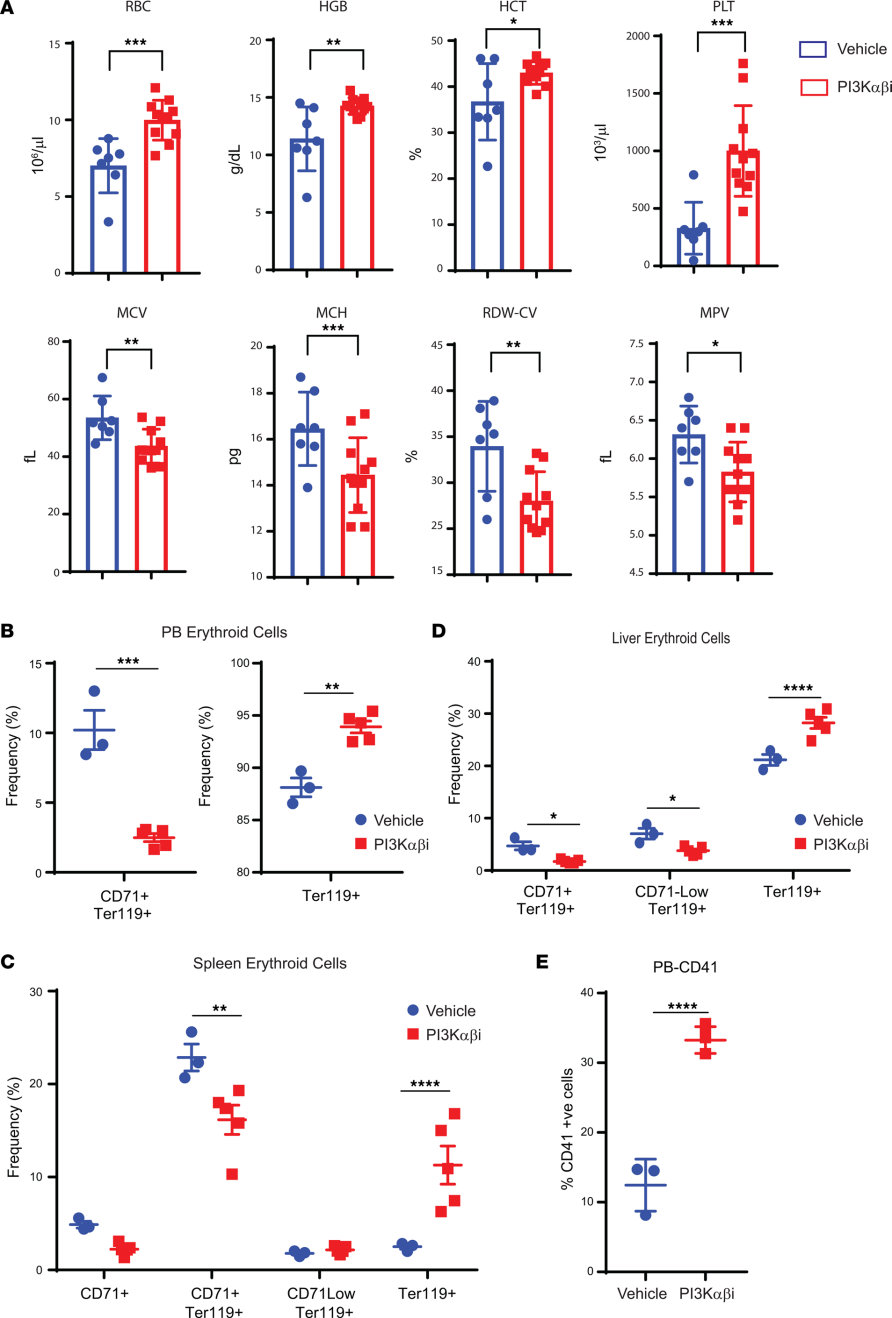
PI3K αβ inhibition improves erythroid cell maturation in mice bearing *Dnmt3a^–/–^* malignant cells. (**A**) Peripheral red cell parameters in mice transplanted with *Dnmt3a^–/–^* BM cells treated with vehicle or the PI3K αβ inhibitor (Bay1082439) for 21 days. *n* = 7–11, mean ± SEM, *P = 0.05, **P = 0.005, ***P = 0.0005. (**B**–**D**) Flow cytometry analysis was performed on PB (**B**), spleen (**C**), or liver cells (**D**) from mice transplanted with *Dnmt3a^–/–^* cells and treated with the PI3K αβ inhibitor as in **A**. Quantitative data showing CD71^+^Ter119^+^ immature erythroid cells, CD71^lo^Ter119^+^ immature erythroid cells, and Ter119^+^ mature erythroid cells in vehicle- and PI3K αβ inhibitor–treated mice. *n* = 3–5, mean ± SEM, *P = 0.05, **P = 0.005, ***P = 0.0005, ****P = 0.00005. (**E**) Quantitative data showing increased presence of mature platelets in the PB of drug-treated mice compared with controls. *n* = 3–5, mean ± SEM, ****P = 0.00005. Unpaired *t* test (2-tailed) was performed in **A**, **B**, and **E**. Two-way ANOVA (Sidak’s multiple comparison) was performed in **D** and **E**.

**Figure 6:**
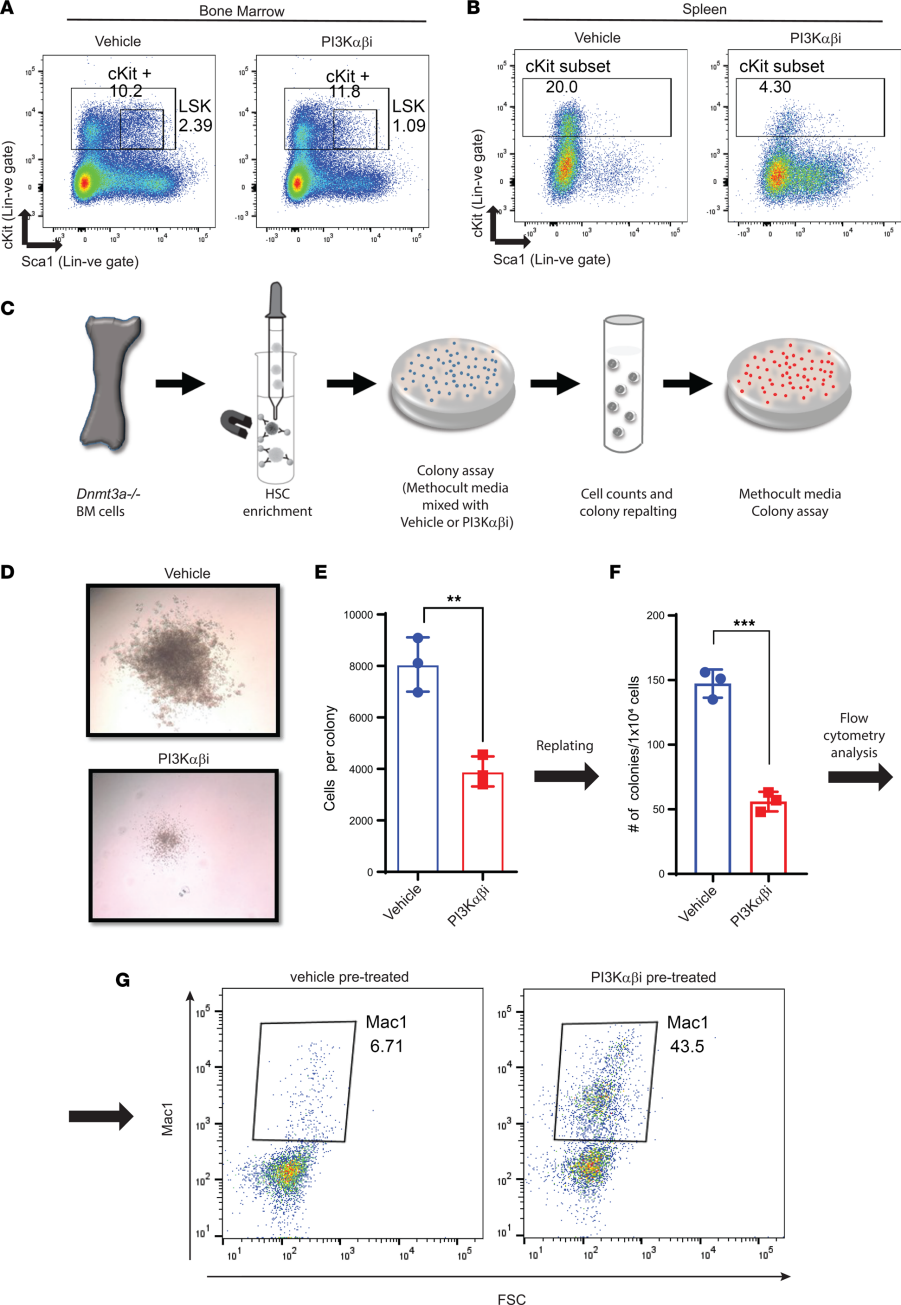
PI3K inhibition promotes cell differentiation in *Dnmt3a-*deleted hematopoietic stem cells. (**A** and **B**) BM and spleen cells from mice transplanted with *Dnmt3a^–/–^* cells and treated with the PI3K αβ inhibitor were subjected to flow cytometry analysis to detect Lin^–^c-KIT^+^Sca-1^+^ cells. Representative dot plots showing Lin^–^c-KIT^+^Sca-1^+^ cells in BM (**A**) and spleen (**B**). (**C**) A schematic depicting our strategy to assess the impact of PI3K inhibitor on the ability of *Dnmt3a^–/–^* HSPCs to give rise to colonies in a methylcellulose-based assay in vitro. Briefly, HSPCs were enriched from the BM of *Dnmt3a^–/–^* mice and platted in a methylcellulose-based media, along with the PI3K inhibitor and cytokines (50 ng/mL rmSCF, 10 ng/mL rmIL-3, 10 ng/mL rhIL-6, 3 U/mL rhEPO, 10 ng/mL Flt-3, and 10 ng/mL thrombopoietin (TPO). Colonies were enumerated on day 7, and cells were replatted in methylcellulose media along with growth factors and colonies were scored again after secondary platting. (**D**) Equal number of cells (10,000 cells) were plated in methocult media and cultured for 1 week in the presence of PI3Kαβ inhibitor (250 nM) or vehicle. Representative colony images depicting reduced colony size under conditions of PI3K αβ inhibitor treatment compared with vehicle conditions. Experiment was performed in triplicate. Number of colonies and total number of cells per plate were quantified. (**E**) Quantitative data show average number of cells per colony in drug and vehicle treatment groups. *n* = 3, mean ± SD, **P = 0.005. (**F**) Quantitative data showing number of colonies after replatting cells derived from the vehicle- and drug-treated groups. *n* = 3, mean ± SD, ***P = 0.0005. (**G**) Flow cytometry analysis of secondary replatted colonies using an antibody against Mac1. Dot plots show the level of Mac1 expression in drug-treated versus vehicle-treated groups. Experiment was performed in triplicate. Unpaired *t* test (2-tailed) was performed in **E** and **F**.

Loss of *Dnmt3a* results in myeloid cell infiltration into the liver, along with impaired erythroid cell maturation and reduced sinusoid spaces, as assessed by histological analysis (13). We next did similar histological analysis on vehicle- and drug-treated livers and spleens of mice bearing *Dnmt3a^–/–^* cells and observed that vehicle-treated liver tissues resemble those of published data (13) (Figure 3H). In the vehicle-treated mice, the main gross changes included severe splenomegaly, hepatomegaly, and hypercellular BM, consistent with increased metamyelocytes in the spleen and BM (Figure 3). Morphological analysis of cytospins revealed increased metamyelocytes and myeloblasts with dysplastic features and hypersegmented neutrophils. The PB smears revealed hypercellularity and hypersegmentation (Figure 3E). Histological observation of spleen sections from vehicle-treated mice showed disarray of normal splenic architecture with a reduction and almost total absence of the white pulp in some areas and increased red pulp area with extramedullary hematopoiesis and focal scattered areas of myeloid dysplasia (Figure 3I). Scattered dysplastic megakaryocytes were observed in the spleen of vehicle-treated mice. Furthermore, the liver was characterized by numerous infiltrating lymphocytes distending the sinusoids throughout the liver parenchyma (both portal and lobular) with scattered focal areas of necrosis, areas of fibroplasia, and hemorrhage. BM cellularity was hypercellular with increased focal areas of myeloid dysplasia (metamyelocytes) throughout the BM in the vehicle group (Figure 3G). In the PI3K inhibitor–treated mice, the liver hepatocytes had a ground glass appearance in the cytoplasm, and some had cytoplasmic vacuolation (Figure 3H). The spleen of the drug-treated mice had forming germinal centers and prominent smooth muscle bands extending down from the splenic capsule, reflecting restoration of splenic architecture (Figure 3I). Fewer areas of myeloid dysplasia, mainly metamyelocytes in the red pulp area and reduced myeloblasts underneath the capsule of splenic region in PI3K inhibitor–treated mice compared with vehicle-treated mice, were seen (Figure 3I). We also observed decreased myeloblast cells and metamyelocytes in the BM cytospin analysis of drug-treated mice compared with the vehicle group (Figure 3F). In addition, BM cellularity was normocellular, with few dysplastic megakaryocytes compared with the vehicle group (Figure 3G). In the myeloid cell compartment of the BM, a reduction in the frequency of myeloid progenitors (Lin^–^cKit^+^ cells), including GMPs, was noted in mice treated with the PI3K α/β inhibitor compared with vehicle controls (Figure 4, A and B). Consistent with an increase in erythroid and platelet progenitors seen in drug-treated mice in Figure 5, drug treatment also led to an increase in the frequency of MEPs in the BM (Figure 4B). In the spleen, the frequency of cKit^+^ cells within the Lin^–^cKit^+^Sca-1^+^ fraction was significantly reduced in drug-treated mice, with a concomitant increase in the frequency of Sca-1^+^ cells (Figure 4, C and D, and Figure 6B). Consistently, a similar reduction in myeloid cells in the spleen of drug-treated mice was observed compared with controls (Figure 4, E and F). No difference in the frequency of B or T cells in the spleen of drug-treated mice was observed (Figure 4, G and H).

The observed restoration of erythroid cells in drug-treated *Dnmt3a^–/–^* mice was intriguing, which led us to examine this in more detail. As seen in Figure 5A, PB erythroid parameters including RBCs, HCT and platelet counts were partially restored in *Dnmt3a^–/–^* mice treated with the drug. These findings were further validated by flow cytometry analysis examining the composition of erythroid progenitors in the PB, spleen, and the liver of drug-treated mice. An increase in the presence of differentiated erythroid progenitors was observed in all 3 tissues, as reflected by the presence of increased frequency of Ter119^+^ cells (Figure 5, B–D). A similar increase in the frequency of platelets was noted in drug-treated mice in the PB (Figure 5E).

**Figure 7:**
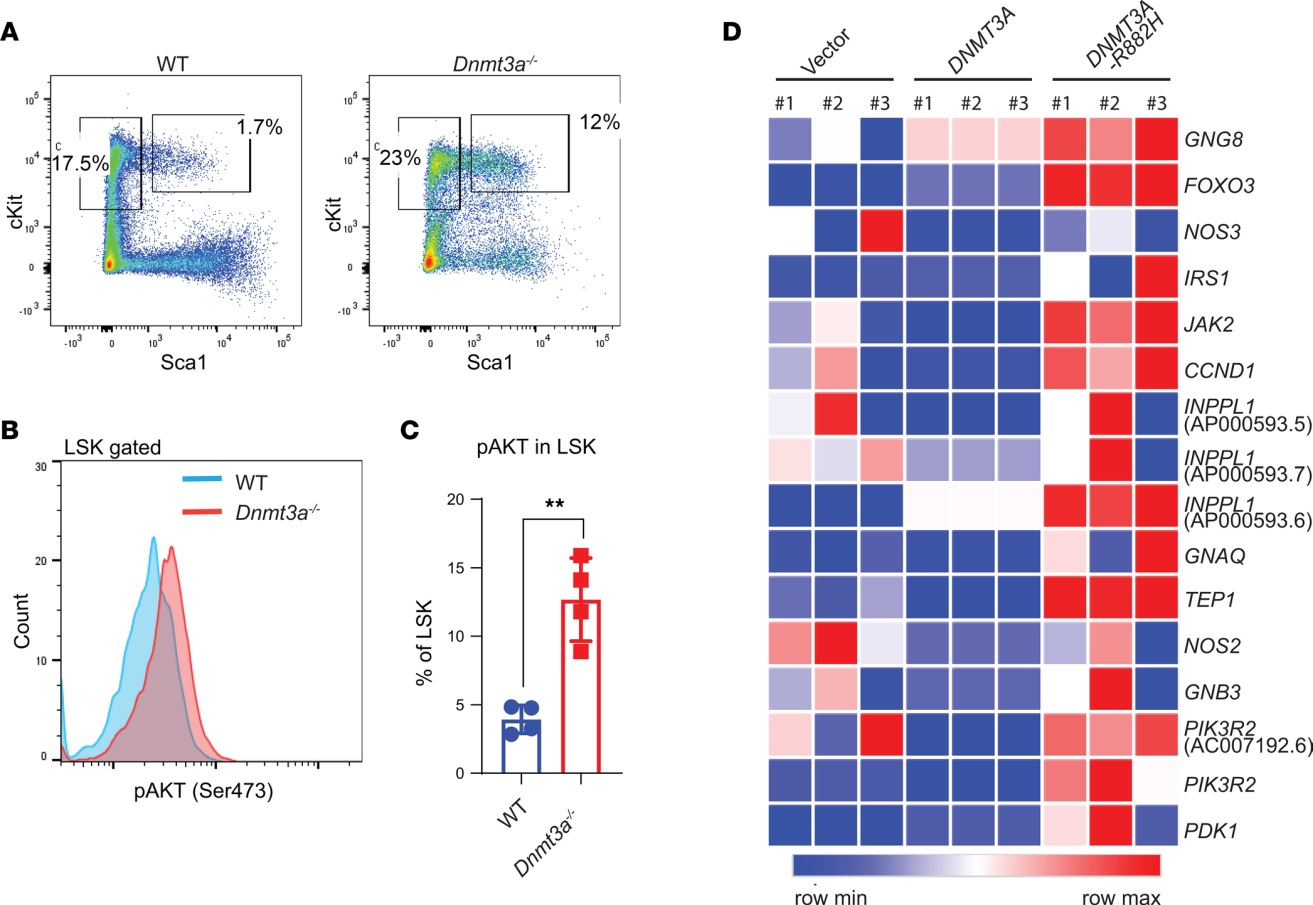
Increased PI3K signaling in *Dnmt3a*-deleted hematopoietic LSK cells and *DNMT3A*-mutated hCD34^+^ HSPCs. (**A**) Flow cytometry analysis performed on BM cells collected from malignant *Dnmt3a^–/–^* and age-matched WT mice. Representative plots for Lin^–^Sca-1^+^cKit^+^ cells from the indicated genotypes are shown. (**B**) Histogram showing intracellular activation of Akt (Ser473) in LSK cells from indicated genotypes. (**C**) Quantitative data showing percent phospho-AKT^+^ LSKs relative to IgG controls. *n* = 4, mean ± SEM, unpaired *t* test (2-tailed), **P = 0.005. (**D**) CB CD34^+^ HSC/Ps were transduced with empty vector, *DNMT3a*-GFP, or *DNMT3aR882H*-GFP, and sorted GFP^+^ cells were subjected to RNA-Seq. Heatmap showing PI3K pathway related gene expression.

To assess the impact of PI3K α/β inhibition at a more primitive HSC/P level, we performed flow cytometry analysis on the BM and spleens of vehicle- and drug-treated mice. We found a decrease in the frequency of Lin^–^cKit^+^Sca-1^+^ cells in the BM and in the spleen of drug-treated mice compared with controls (Figure 6, A and B). Given the decrease in the frequency of primitive *Dnmt3a^–/–^* cells upon drug treatment as well as the enhanced differentiation of these cells, we assessed the colony-forming ability in an in vitro methylcellulose assay. Figure 6C shows a schematic depicting the in vitro experimental strategy. As seen in Figure 6, D–F, drug treatment reduced the colony size and the number of cells per colony upon primary plating and decreased the number of total colonies upon secondary plating (Figure 6, C–E). In addition to reduction in the number of cells per colony, an increase in the differentiation of stem/progenitor cells was observed, as reflected by the presence of an increase in the frequency of Mac1^+^ cells, as assessed by flow cytometry (Figure 6, F and G). All of these changes were associated with increased AKT activation in *Dnmt3a*-null LSK cells (Figure 7, A–C). We also examined if some aspect of PI3 kinase signaling was modulated in human HSC/Ps bearing a *DNMT3A* mutation. We transduced and sorted cord blood CD34^+^ cells bearing either the empty vector or WT *DNMT3A*-GFP or *DNMT3A R882H*-GFP and subjected these cells to RNA-Seq analysis. As shown in Figure 7D, an increase in the expression of several genes associated with the PI3 kinase pathway was observed in mutant-bearing cells compared with controls.

**Figure 8:**
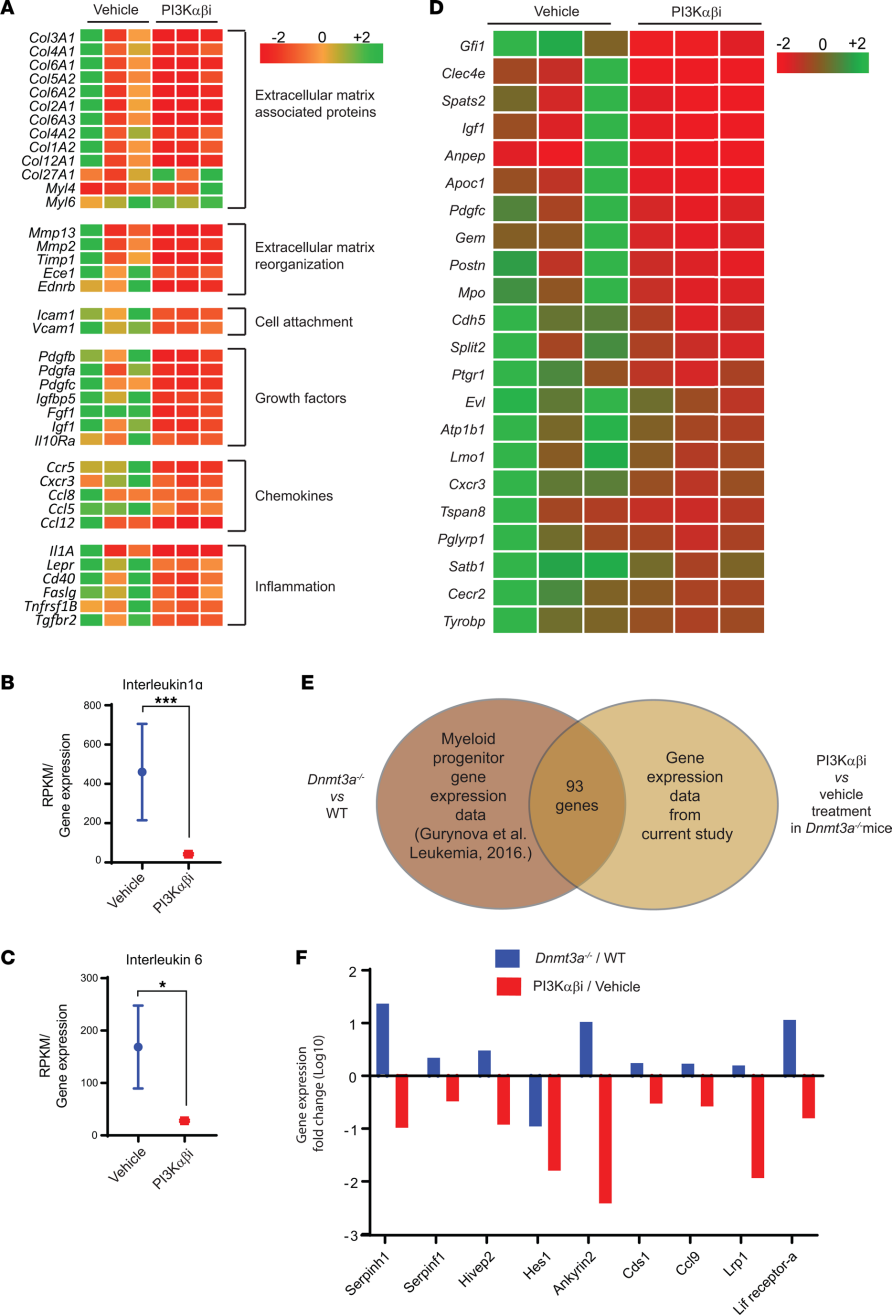
PI3K inhibition reverts *Dnmt3a* loss–induced changes in the expression of genes involved in cell migration and inflammation and alters the expression of fetal liver HSC genes. BM cells were collected from vehicle or PI3K αβ inhibitor–treated mice bearing *Dnmt3a*^–/–^ malignant cells. RNA was isolated and subjected to next-generation sequencing, and gene expression analysis was performed. (**A**) Heatmap showing that PI3K αβ inhibitor treatment reduces the expression of genes involved in cell motility, cell attachment, inflammatory cytokines, and chemokines. (**B** and **C**) Quantitative assessment of the level of expression of IL-1α and IL-6 in drug-treated mice versus controls. *n* = 3, mean ± SEM, EdgeR DE analysis, *P = 0.05, ***P = 0.0005. (**D**) Heatmap showing gene expression involved in the development of fetal liver HSCs downregulated as a result of PI3K αβ inhibitor treatment in the *Dnmt3a^–/–^* cells versus controls. (**E** and **F**) Analysis of GMP progenitor gene expression data from a study involving *Dnmt3a^–/–^* and WT mice (13) was compared with the BM-derived gene expression data in the current study from vehicle- and PI3K αβ inhibitor–treated mice bearing *Dnmt3a^–/–^* cells. Venn diagram shows that 93 genes were found to be differentially regulated in both data sets. Comparative assessment of gene expression fold changes of indicated genes (**F**) in *Dnmt3a^–/–^/*WT and PI3Kαβ/vehicle treatment groups.

Next, we performed RNA-Seq studies on BM cells derived from drug-treated and control mice. Gene expression analysis showed that genes involved in tumor invasion, cell motility, migration, and attachment were all repressed in the drug-treated mice compared with vehicle control (Figure 8A). Likewise, a reduction in the expression of growth factor–encoding genes as well as chemokines and genes involved in inflammation including IL-6 and IL-1α were downregulated in drug-treated mice (Figure 8, A–C). Guryanova et al. reported that *Dnmt3a^–/–^* mice show liver specific HSC expansion (13). Fetal liver HSCs exhibit robust self-renewal capacity and long-term multilineage reconstitution potential and share features seen in MDS/MPN (13, 29). Loss of *Pten* during fetal-to-adult hematopoiesis causes sustained fetal hematopoiesis resulting in the development of myeloid leukemia (30). To verify if PI3K α/β inhibitor treatment modulates the fetal liver–specific HSC gene signature seen in *Dnmt3a^–/–^* mice, we compared our gene expression data with the fetal liver HSC gene expression data reported earlier (31). As seen in Figure 8D, PI3K α/β inhibitor represses the fetal liver HSC gene expression signature seen in *Dnmt3a^–/–^* cells compared with vehicle-treatment group.

**Figure 9:**
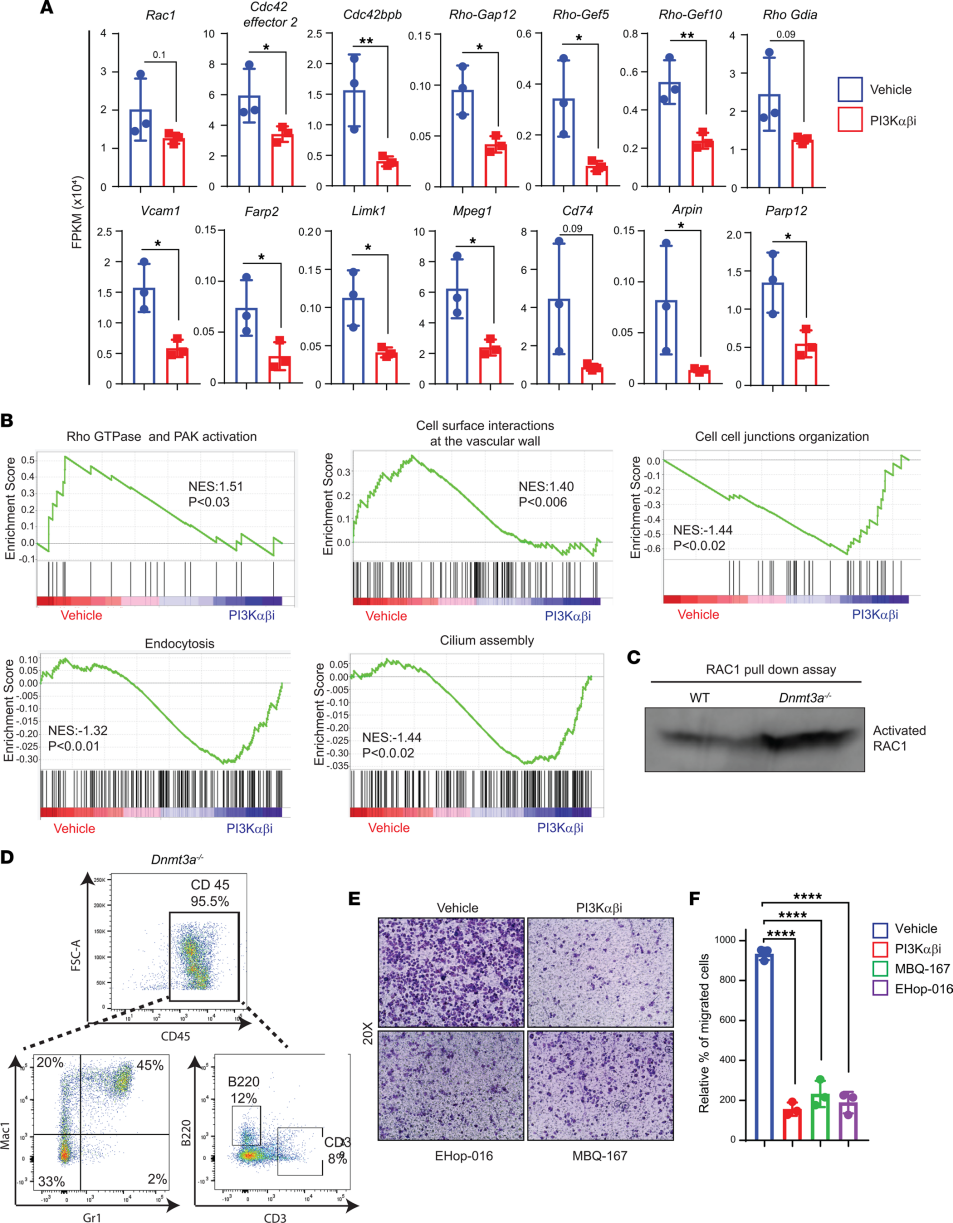
PI3K signaling promotes *Dnmt3a* loss–induced cell migration via RAC1 GTPase. (**A**) Quantitative assessment of the level of expression of genes that belong to the RAC1/CDC42 pathway that are significantly reduced in PI3K drug–treated group compared with vehicle group. *n* = 3, mean ± SD, unpaired *t* test (2-tailed), *P = 0.05, **P = 0.005. (**B**) Gene set enrichment plots showing reduced RHO GTPase and PAK signaling associated with RAC1 pathway and altered biological processes in PI3K αβ inhibitor–treated *Dnmt3a^–/–^* BM cells compared with vehicle-treated group. (**C**) BM cells collected from WT or *Dnmt3a^–/–^* mice were subjected to activated RAC1 pulldown assay using PAK binding domain beads. Western blot analysis was performed to detect activated RAC1 using an anti-RAC1 specific antibody. Representative of 3 independent experiments shown. (**D**) Liver tissues harvested from hepatomegaly presenting malignant *Dnmt3a^–/–^* mice were subjected to CD45^+^ cell enrichment using magnetic cell separation, and percent CD45 enrichment was assessed using flow cytometry. Figure shows the percent of Mac1^+^, Gr1^+^, B220^+^, and CD3^+^ cells within the CD45^+^ enriched fraction of *Dnmt3a^–/–^* cells. (**E** and **F**) CD45^+^ cells (2.5 × 10^5^) enriched from *Dnmt3a^–/–^* mice livers as in **D** were subjected to transwell migration assay for 20 hours at 37°C in the presence or absence of 100 nM PI3K αβ inhibitor (Bay1082439), 0.5 μM RAC1/3 inhibitor (EHop-016), or 200 nM RAC/CDC42 inhibitor (MBQ-167). Migrated cells were stained with 0.1% crystal violet, and representative images (20***×*** magnification) for treatments are shown. Quantitation of percent migrated cells is shown in **F**. *n* = 3, mean ± SD, 1-way ANOVA (Tukey’s multiple comparison test), ****P < 0.00001.

As noted above, PI3K inhibition in mice bearing *Dnmt3a*^–/–^ cells significantly inhibits the infiltration of myeloid cells in various tissues. *Pten* negatively regulates PI3K pathway and is required to maintain quiescence in HSCs; however, PTEN loss results in HSC expansion, mobilization, and impaired BM retention via RAC signaling (37, 38). PTEN regulates HSC mobilization via a cell-autonomous mechanism (39). We checked if PI3K signaling components are involved in *Dnmt3a* loss–induced HSC/P egress and extramedullary hematopoiesis. Gene expression analysis showed reduced expression of genes involved in cell motility, mostly pertaining to the RHO/RAC/CDC42 pathway in PI3K drug-treated mice relative to controls (Figure 9A). These include *Rac1*, *Cdc42se2*, *CDC42bp*, *Rho-GAP 12*, *Rho-GEF10*, *Rho-GEF5*, and *Rho Gdia*. Expression of key genes related to cell attachment and cell invagination such as *Vcam1*, *Frap2*, *Limk1*, *Arpin*, and *Cd74* were repressed in PI3K inhibitor–treated *Dnmt3a^–/–^* mice compared with controls (Figure 9A). The expression of these genes was validated by quantitative PCR (qPCR) on sorted CD45.2^+^ cells (*Dnmt3a^–/–^*) from vehicle- or PI3Kαβ inhibitor–treated F1 mice (Figure 2A and Supplemental Figure 2B; supplemental material available online with this article; https://doi.org/10.1172/jci.insight.163864DS1). VCAM1 is known for leukocyte-endothelial cell adhesion via interaction with integrins and activates calcium release and RAC1 activation (40). Arpin (ARP2/3 complex inhibitor protein) regulates actin polymerization at the lamellipodium tip in response to RAC signaling (41). Similarly, FARP2 (Rho GEF and pleckstrin domain protein 2) acts as a guanine nucleotide exchange factor that activates RAC1 and regulates actin polymerization (42). CDC42 binding protein targets LIMK1 to lamellipodium in F-actin regulation. Rho GEF5 plays a role in actin polymerization involving Src and PI3K activation (42). Rho GDIα retains CDC42, RAC1, and RHOA in inactive cytosolic pool and keeps it from degradation (43). MPEG1 (macrophage expressed gene 1) inhibits the acidification of phagocytic vacuole (44). Blockade of CD74 receptor or its ligand macrophage migration inhibitory factor (MIF) reduces cell invagination (45). Collectively, we see increased gene enrichment for RHO GTPase pathway in *Dnmt3a^–/–^* cells treated with vehicle compared with PI3K inhibitor (Figure 9B). Furthermore, increased gene enrichment for the RHO GTPase pathway and leukocyte extravasation related cell-surface-interaction-at-vascular-wall was seen in the *Dnmt3a^–/–^* vehicle group compared with the inhibitor treatment group (Figure 9B). In contrast, cilium assembly formation, which points to cell cycle exit to differentiation cue, endocytosis, and cell-to-cell junction–related gene enrichment, occurred less in the vehicle-treated group compared with PI3K inhibitor–treated group (Figure 9B). Selective enrichment and detection of GTP-bound RAC1 GTPase through specific protein interaction with the PAK1 protein-binding domain showed increased activated RAC1 in BM cells from malignant *Dnmt3a^–/–^* mice compared with WT mice (Figure 9C). To determine if inhibition of the RAC/CDC42 pathway affects the ability of liver-specific hematopoietic cells to migrate and invaginate, CD45^+^ cells enriched from *Dnmt3a^–/–^* mouse liver tissues were subjected to a transwell migration assay in the presence or absence of PI3K or RAC inhibitors. Consistent with prior studies, we also found abundant CD45^+^ hematopoietic cells in *Dnmt3a^–/–^* mouse liver tissues compared with *Dnmt3a^+/+^* mice (13). CD45-gated cells from *Dnmt3a^–/–^* liver tissues showed mostly myeloid cells and few lymphoid lineage cells (Figure 9D). PI3K αβ inhibitor (100 nM Bay1082439), Rac1/3 inhibitor (0.5 μM EHop-016), or RAC/CDC42 inhibitor (200 nM MBQ-167) treatment significantly reduced the migration of liver-specific *Dnmt3a^–/–^* hematopoietic cells compared vehicle treatment (Figure 9, E and F).

**Figure 10:**
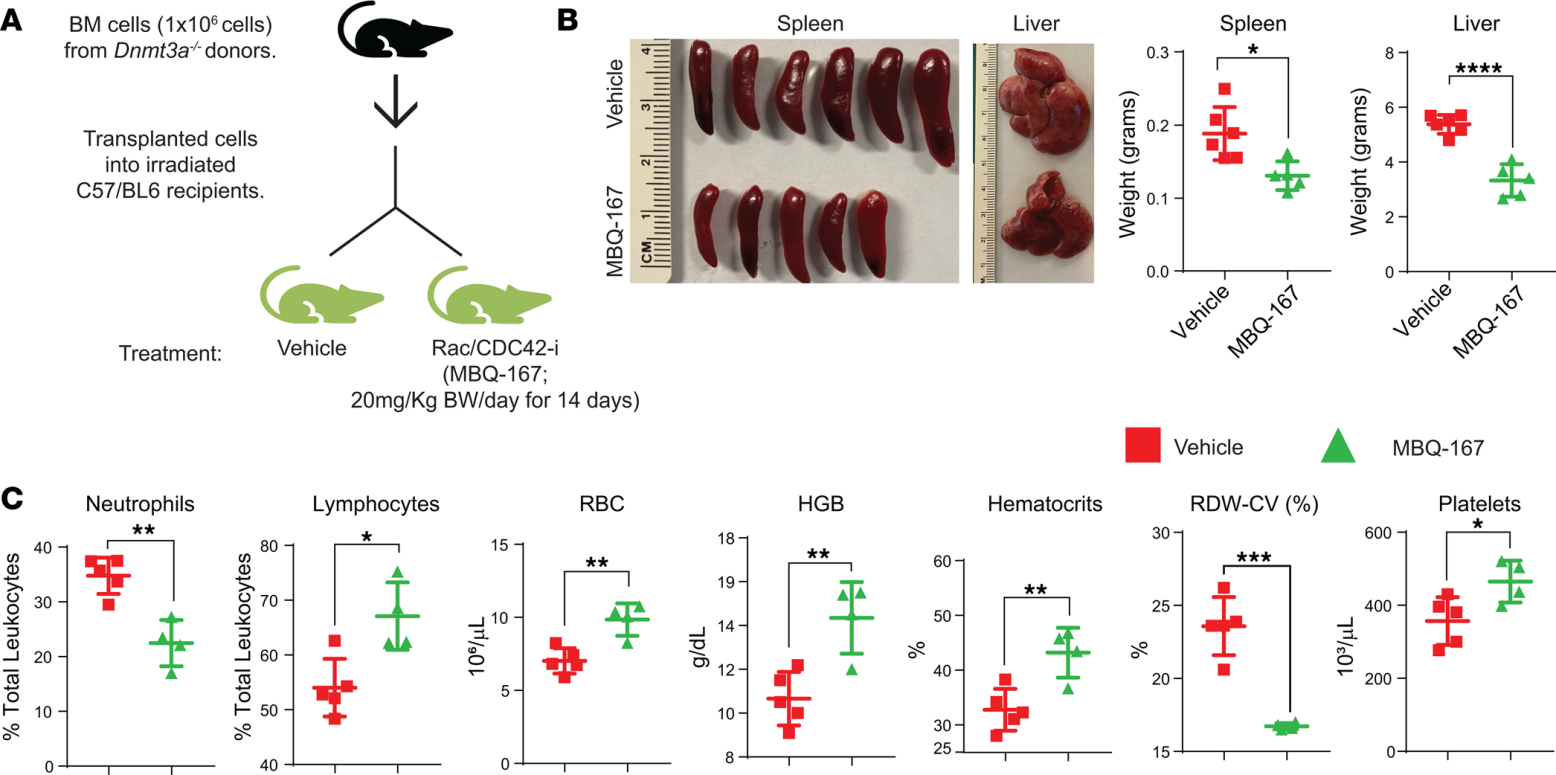
RAC/CDC42 pathway inhibition partially rescues hepatosplenomegaly and decreases *Dnmt3a* loss–induced myeloid malignancy characteristics in mice. (**A**) BM cells from *Dnmt3a^–/–^* mice were transplanted into lethally irradiated C57BL/6 mice, and 6 weeks after transplantation, mice were treated with vehicle or the RAC/CDC42 inhibitor (MBQ-167; 20 mg/kg body weight/day) for 14 days. (**B**) Images of spleens and livers from the vehicle- and drug-treated mice. Quantitative assessment of liver and spleen weights from vehicle- or drug-treated mice. *n* = 5–6, mean ± SEM, *P < 0.05, ****P < 0.00005. (**C**) Peripheral red cell parameters in mice in **A**. *n* = 4–6, mean ± SEM, *P = 0.05, **P = 0.005, ***P = 0.0005, unpaired *t* test (2-tailed) performed (**B** and **C**).

**Figure 11:**
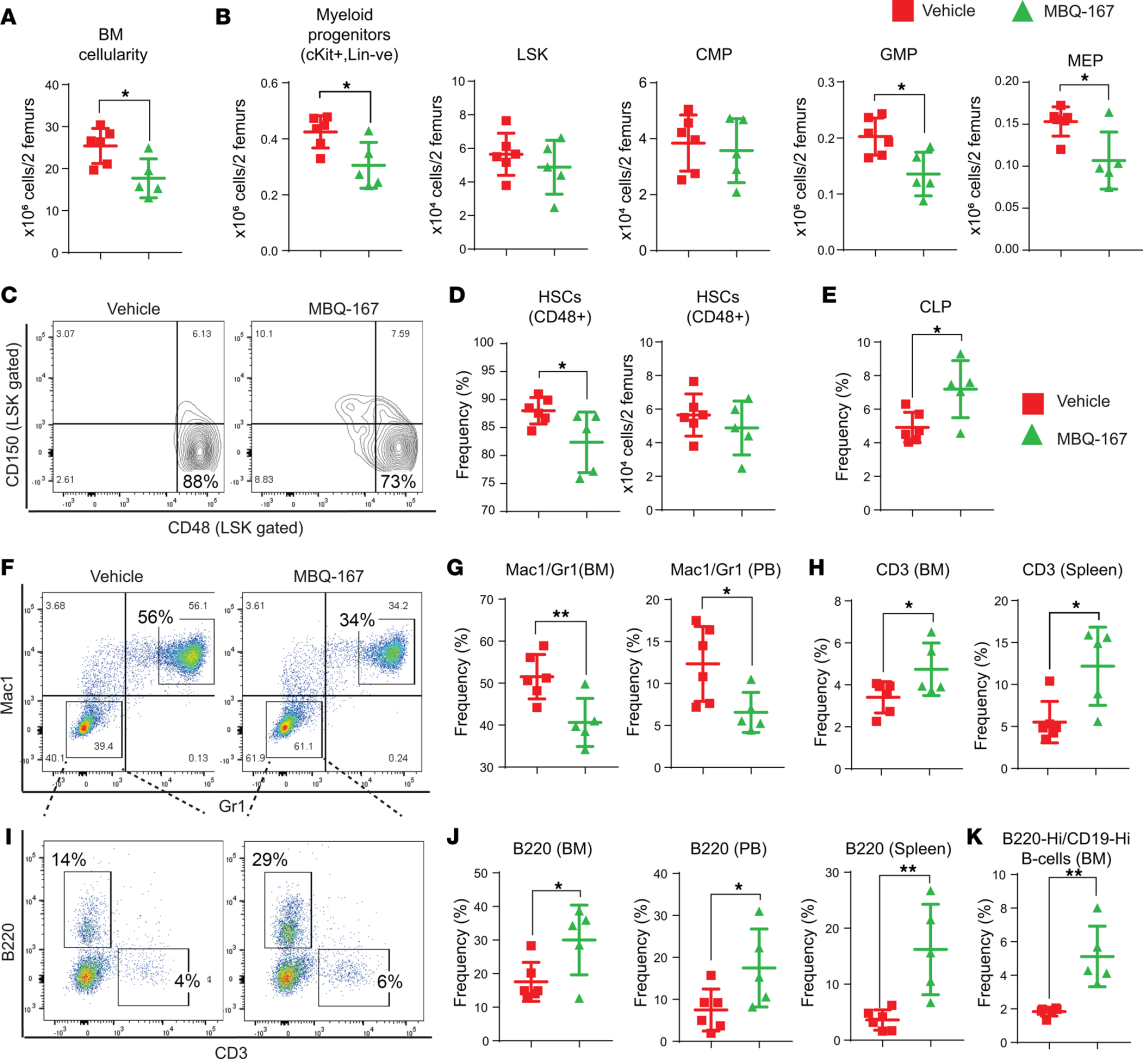
RAC/CDC42 pathway inhibition decreases *Dnmt3a* loss–induced myeloid skewing and improves B cells and T cells in mice. (**A**) BM cellularity quantitation for 2 femurs in each mouse from mice transplanted with *Dnmt3a^–/–^* cells and treated with the vehicle or MBQ-167. *n* = 5–6, mean ± SEM, *P = 0.05. (**B**) Flow cytometry analysis was performed on BM cells from mice transplanted with *Dnmt3a^–/–^* cells and treated with vehicle or MBQ-167. Quantitative data showing the absolute number of Lin^–^cKit^+^ myeloid progenitors, Lin^–^cKit^+^Sca-1^+^ (LSKs), CMPs, GMPs, and MEPs. *n* = 5–6, mean ± SEM, *P = 0.05. (**C** and **D**) Flow cytometry analysis was performed on BM cells from mice in **A**, and representative dot plots for CD150- and CD48-stained (LSK gated) HSCs as shown in **F**; quantitative data plotted for the frequency and absolute number of CD150^–^CD48^+^ cells is shown in **G**. *n* = 5–6, mean ± SEM, *P = 0.05. (**E**) Representative data showing the frequency of CLPs in BM cells from mice transplanted with *Dnmt3a^–/–^* cells and treated with vehicle or MBQ-167. *n* = 5–6, mean ± SEM, *P = 0.05. Flow cytometry was performed on BM cells from vehicle- or MBQ-167–treated mice as in Figure 10A, and a representative dot plot for Mac1/Gr1 expression is shown in **F** and B220/CD3 cells in **I**. Quantitative data showing decreased Gr1^+^Mac1^+^ in BM and PB fractions (**G**); increased CD3^+^ T cells in BM and spleen fractions (**H**); increased B220^+^ B cells in BM, spleen, and in PB fractions (**J**); and increased B220^hi^CD19^hi^ mature B cells in BM fractions (**K**) of drug-treated mice compared with controls. *n* = 5–6, mean ± SEM, *P = 0.05, **P = 0.005, unpaired *t* test (2-tailed) performed (**B**, **D**, **E**, **G**, **H**, **J**, and **K**).

Given the increased activation of RAC as well as reduced migration of *Dnmt3a*-null cells upon RAC/CDC42 inhibition, we investigated the role of RAC/CDC42 pathway in vivo in mice bearing *Dnmt3a^–/–^* cells using a RAC/CDC42 inhibitor (MBQ-167) (Figure 10A). RAC/CDC42 inhibition significantly reduced hepatosplenomegaly when compared with vehicle group (Figure 10B). Similar to the response seen with the PI3K inhibition in *Dnmt3a*-null cell–bearing mice, PB analysis of RAC/CDC42 inhibitor–treated *Dnmt3a*-null mice showed reduced neutrophil percentages, increased lymphoid cell percentages, and increased RBCs, hemoglobin, hematocrits, and platelets compared with vehicle control group (Figure 10C). High red cell distribution width–coefficient of variation percentage (RDW-CV%) is a known indicator of leukemia-associated anemia. We observed a marked decrease in RDW-CV% in RAC/CDC42 inhibitor–treated mice compared with vehicle group (Figure 10C). RAC/CDC42 inhibitor decreased BM cellularity and absolute number of myeloid progenitors (c-KIT^+^/Lin^–^), GMPs, and MEPs but not CMPs compared with vehicle group (Figure 11, A and B). MBQ-167 treatment also decreased the absolute number of CD48^+^ HSCs compared with vehicle group (Figure 11, C and D). RAC/CDC42 signaling inhibition improved CLP frequencies, decreased Mac1^+^/Gr1^+^ myeloid cells, increased CD3^+^ T cells in the BM and PB fractions of drug-treated mice, and increased B cell frequencies in BM, spleen, and PB fractions (Figure 11, E–K). RAC/CDC42 inhibitor treatment also partially restored *Dnmt3a* loss–induced erythroid maturation defect in the BM and spleen, similar to the PI3K inhibitor treatment (Supplemental Figure 3). Taken together, these results demonstrate that, downstream from PI3K, the RAC/CDC42 pathway plays an essential role in *Dnmt3a* loss–induced pathological changes in HSC/Ps.

**Figure 12:**
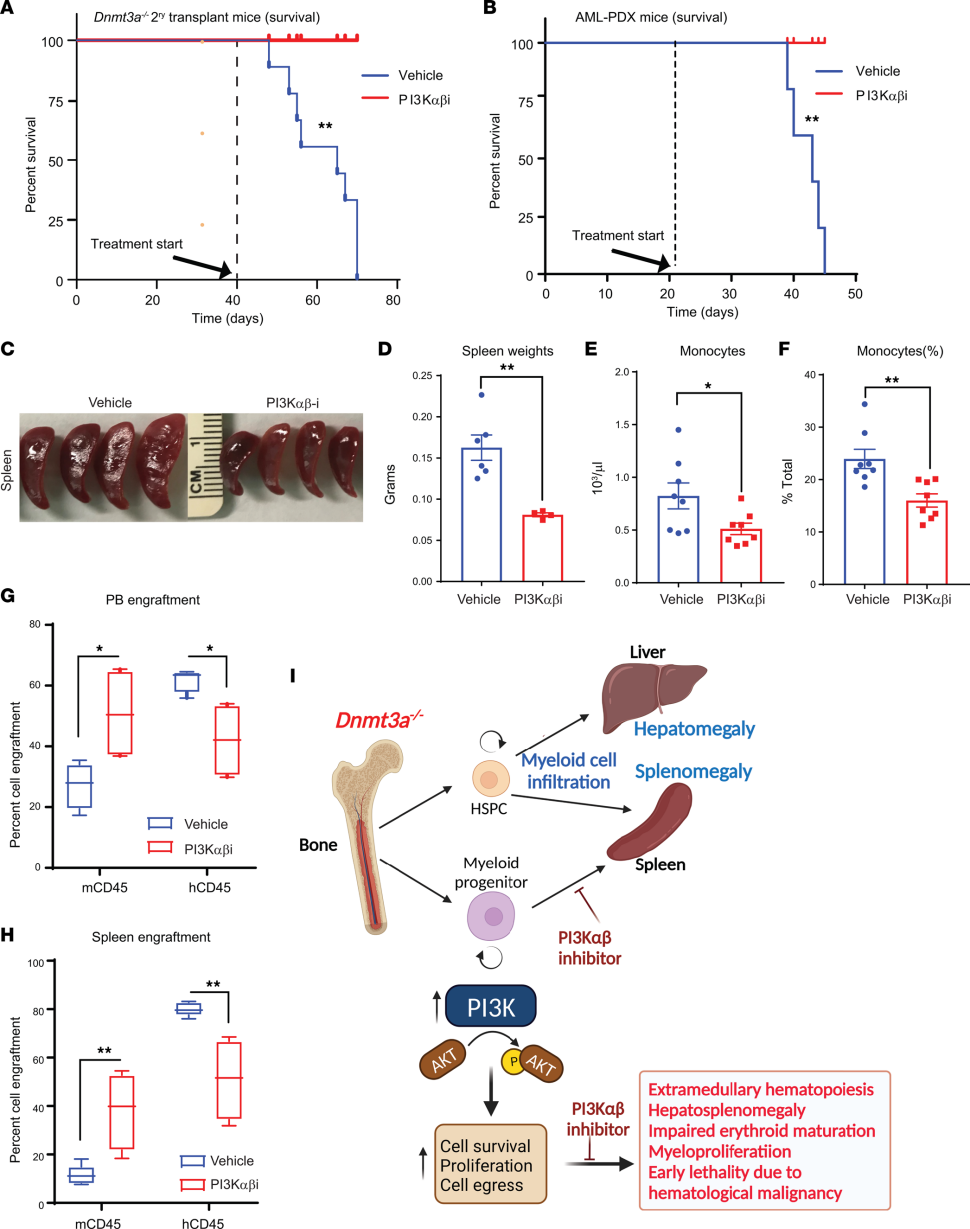
PI3K inhibitor treatment of mice bearing human AML cells bearing *DNMT3A* mutation enhances their survival. (**A**) Secondary recipients of *Dnmt3a^–/–^* BM cells were prepared for the survival study by injecting 2 ***×*** 10^6^ of *Dnmt3a^–/–^* cells from primary BM transplants of *Dnmt3a^–/–^* cells. Six weeks after transplantation, these mice were treated with the PI3K inhibitor (Bay1082439) for 21 days (75 mg/kg body weight). As seen in **A**, all mice belonging to the vehicle group succumbed by day 70 after transplant, whereas none of the drug-treated mice died within this time period. *n* = 9, log-rank (Mantel-Cox) test, *P* < 0.003. (**B**) Patient-derived AML cells (1 million) bearing *DNMT3A* mutation were transplanted into NSG-cyto mice to generate AML-PDX. Starting day 20 after transplant, these mice were treated with vehicle or PI3K αβ inhibitor for 21 days. As seen in **B**, all mice belonging to the vehicle group succumbed by day 45 after transplant, whereas none of the drug-treated mice died within this time period. *n* = 5, log-rank (Mantel-Cox) test, *P* < 0.001. (**C**) Images of spleens derived from drug- and vehicle-treated mice. (**D**) Quantitative analysis of spleen weights from vehicle- and drug-treated AML-PDX mice. *n* = 5, mean ± SEM, unpaired *t* test (2-tailed), *P* < 0.001. (**E** and **F**) Quantitative analysis of the number of monocytes in vehicle- versus drug-treated mice. *n* = 8, mean ± SEM, unpaired *t* test (2-tailed), *P = 0.05, **P = 0.005. (**G** and **H**) Quantitative assessment of percent engraftment of murine and human CD45 cells in the PB and spleen of vehicle- and drug-treated mice as assessed by flow cytometry. *n* = 5–8, 2-way ANOVA, *P = 0.05, **P = 0.005. The boxes shown with lower and upper quartiles separated by the median (horizontal line), and the whiskers extend to the minimum and maximum values. (**I**) Model illustrating the role of PI3K signaling in *Dnmt3a* loss–induced myeloid malignancy. PI3Kαβ specific inhibitor blocks the *Dnmt3a* loss–induced malignant characteristics and improves survival.

While the above observations suggest a role for the PI3K pathway in loss of *Dnmt3a*–induced hematopoietic abnormalities, we next examined whether human AML cells carrying a *DNMT3A* mutation respond to PI3K α/β inhibitor treatment in a manner similar to murine mutant cells. We transplanted *DNMT3A* mutation–bearing AML cells into NSG-cyto mice and waited for these mice to engraft human cells. After successful engraftment of hCD45 cells, drug treatment was initiated for 21 days utilizing the PI3K α/β inhibitor. As seen in Figure 12B, while all vehicle-treated mice succumbed to death by day 45, none of the drug-treated mice were found dead at this time point. Figure 12C shows reduced spleen sizes in drug-treated leukemic mice, and Figure 12D provides a quantitative reduction in the spleen weights of drug-treated mice. A significant reduction in total number of monocytes as well as in the frequency of monocytes was observed in drug-treated mice (Figure 12, E and F). Importantly, both the PB and splenic engraftment of human leukemic CD45 cells was significantly reduced in the drug-treated mice as opposed to vehicle-treated mice with a concomitant increase in the detection of WT murine CD45 cells (Figure 12, G and H). A similar rescue in the survival of murine *Dnmt3a^–/–^* cell–bearing mice treated with the PI3K inhibitor as that seen in the PDX transplanted model was observed (Figure 12A) (13).

## Discussion

Given that 25% of AML and about 10% of MPN and MDS bare *DNMT3A* mutations, which are associated with increased risk of relapse and poor overall prognosis; being able to target DNMT3A and downstream signals from DNMT3A is a prudent therapeutic approach. However, in spite of some understanding of how DNMT3A mutations induce pathological changes and transform cells, an understanding of how to target this mutation or its downstream consequences remain poorly understood. To this end, treatment of patients with AML with *DNMT3A* mutations using hypomethylating agents (HMA) such as azacitidine and decitabine is being used in the elderly with comorbidities. A few studies using this approach have shown positive outcomes in patients with AML and patients with MDS with *DNMT3A* mutations, although the mechanisms are poorly understood (46–48). Others have begun to use a combination of HMAs with other targeted agents and have reported good outcomes in patients with *DNMT3A* mutations (49). Additional studies have shown a utility for using ibrutinib in AML with *FLT3-ITD*, *NPM1*, and *DNMT3A* mutations (50) as well as using JAK1/2 kinase inhibitor ruxolitinib plus venetoclax or with BET inhibitors (51, 52). Furthermore, downregulation of HOX genes in *DNMT3A* mutant AML using DOT1 inhibitors has also been demonstrated (53). While intriguing, significant number of these studies will need to be validated in a preclinical setting. Our studies utilizing a PI3K inhibitor against the α and β catalytic subunits of PI3K provides a strong rationale for targeting the PI3K pathway for the treatment of myeloid malignancies bearing *DNMT3A* mutations. Our studies show that PI3K pathway plays a crucial role in *Dnmt3a* loss–driven myeloid malignancy in multiple places during the progression to leukemia (Figure 12I).

Previous studies have shown that diseased *Dnmt3a*^–/–^ mice present with anemia, reduced mature erythroblasts, and an accumulation of immature CD71^+^Ter119^+^ proerythroblasts in the spleen and in the BM (13). Our results suggest that PI3K inhibitor treatment significantly improves the erythroid maturation defects seen in these mice. Thus, PI3K inhibitor could be used in patients for treating anemia related to chemotherapy or in patients with myeloid disorders such as MDS (54).

The function of macrophages in erythropoiesis is a highly coordinated process involving multiple erythroid developmental stages. Macrophages and erythroblasts form erythroblastic islands via intracellular adhesion molecules (55). CD169^+^ macrophages play a critical role in erythropoiesis under homeostasis and in stress. In our RNA-Seq data, we noticed a reduction in the expression of genes involved in erythrophagocytosis such as *Cd163*, *Emp2*, *Vcam1*, and *Icam1* upon PI3K inhibition in *Dnmt3a*^–/–^ mice. We suspect that PI3K inhibition causes homeostasis in erythropoiesis to occur, prevents excessive erythrophagocytosis, and, thus, allows proper RBC development.

## Methods

### Mice.

*Dnmt3a* conditional KO with floxed sequences (*Dnmt3a^fl/fl^*) have been previously described (56). Likewise, floxed *p85*α–conditional KO mice (*p85*α*^fl/fl^*) have also been previously described (57). *p85*α*^fl/fl^* mice were crossed with *Dnmt3a^fl/fl^* mice to obtain *p85*α*^fl/fl^*:*Dnmt3a^fl/fl^* double KO (DKO) mice. Mx-Cre mice were crossed with the DKO mice to generate hematopoietic specific Cre-recombinase expression in hematopoietic cells. Twelve- to 16-week-old conditional KO mice were i.p. injected with 300 μg of poly I:C 3 times with 48-hour time intervals.

All mice were maintained under specific pathogen–free conditions at the Indiana University Laboratory Animal Research Center.

### PI3K inhibitor and RAC/CDC42 inhibitor treatment in vivo.

BM cells from poly I:C–treated *Dnmt3a^fl/fl^:Mx-1–Cre^–^* or *Dnmt3a^fl/fl^:Mx-1–Cre*^+^ were harvested. 0.5x10^6^ *Dnmt3a^fl/fl^:Mx-1–Cre*^+^ donor (CD45.2; Dnmt3a deleted) BM (BM) cells or 0.5x10^6^ *Dnmt3a^fl/fl^:Mx-1–Cre^–^* donor (CD45.2; control) BM cells were transplanted into irradiated recipient mice along with 1 ***×*** 10^6^ control *BoyJ* (CD45.1) BM cells as depicted in Figure 2A. Chimerism was assessed at every 2 weeks after transplantation. Six weeks after transplantation, at 50% baseline chimerism, mice were daily administered orally with vehicle (0.5 methylcellulose [W/V] and 0.1% Tween 80 [V/V]) or PI3K αβ inhibitor (Bay1082439; MedKoo Biosciences) at dosage 75 mg/kg body weight or PI3K αδ inhibitor at dosage 75 mg/kg body weight (GDC-0941; Selleck Chemicals) for 30 days. The drug was prepared in distilled water containing 0.5 methylcellulose (w/v) and 0.1% Tween 80 (v/v). Whole BM transplants were conducted by performing tail vein injections using 2 ***×*** 10^6^ BM cells from poly I:C–treated malignant *Dnmt3a^fl/fl^:Mx-1–Cre^+^* mice. C57BL/6 mice were lethally irradiated and used as recipients. Six weeks after transplantation, transplanted recipient mice were daily administered orally with vehicle (0.5 methylcellulose [w/v] and 0.1% Tween 80 [v/v]) or the PI3K αβ inhibitor (Bay1082439) at a dosage of 75 mg/kg body weight for 21 days and were analyzed. Recipients who were not treated with the PI3K αβ inhibitor became severely ill within 10–12 weeks after transplantation. RAC/CDC42 pathway inhibitor (MBQ-167) treatment studies were performed on mice transplanted with whole BM cells that were prepared by tail vein injections of 2 ***×*** 10^6^ BM cells from malignant *Dnmt3a^fl/fl^:Mx-1–Cre^+^* mice. MBQ-167 was purchased from MedChemExpress LLC. MBQ-167 was reconstituted in 12.5% ethanol, 12.5% Cremophor EL (CrEL), and 75% PBS (pH 7.4) and administered to mice at 20 mg/KgBW per day for 14 days. Methyl cellulose was purchased from Sigma-Aldrich (catalog M0262), Tween 80 from Thermo Fisher Scientific (catalog BP338), and Creophor EL from MilliporeSigma (catalog 238470).

A multimutational BM sample derived from patients with AML bearing *FLT3^ITD^ (ins46)*, *DNMT3A R882H*, *NPM1 W288 FS12*, and *CHEK2* mutations frozen in DMEM supplemented with 20% FBS and 10% DMSO was thawed, and their viability was determined using Trypan blue staining. Cells were filtered through a 40 μm Cell Strainer to remove cell clumps and debris. Primary transplants were performed by injecting 1 ***×*** 10^6^ viable cells into sublethally irradiated (350G) NSGS (NOD.Cg-Prkdcscid Il2rgtm1Wjl Tg [CMV-IL3, CSF2, KITLG]1Eav/MloySzJ) mice. Primary transplant mice were analyzed for the presence of human cells by staining with an antibody against human CD45 cell surface protein 4 weeks after transplantation. Four to 6 weeks after transplantation, mice were euthanized, BM cells were collected, and 1 million viable cells were injected into sublethally irradiated NSGS mice in a secondary transplant assay. Six weeks after secondary transplantation, mice were administered orally with vehicle or PI3K αβ inhibitor at a dosage of 50 mg/kg body weight for 21 days and subsequently analyzed for human AML cell engraftment.

### Analysis of BM-derived cell growth and proliferation.

BMNC derived from control, single KO (*p85*α^–/–^ or *Dnmt3a^–/–^*), and DKO mice (*Dnmt3a^–/^_;_ p85*α^–/–^) were cultured in the presence of 5 ng/mL of IL-3 for 1 week before subjecting them to a thymidine incorporation assay. Briefly, cells were washed and starved of growth factors and serum in DMEM containing 0.2% BSA for 6–7 hours. In total, 1 ***×*** 10^5^ cells were plated in 96-well plates in DMEM supplemented with 10% FBS and 2% penicillin/streptomycin in the presence or absence of IL-3 or SCF. After 48 hours, cells were harvested using an automated cell harvester and subjected to thymidine update assessment. Penicillin/streptomycin, FBS, and DMEM were purchased from Thermo Fisher Scientific. IL-3 and SCF were purchased from PeproTech.

### Purification and culture of human CB CD34^+^ cells.

Mononuclear cells were isolated from CB (obtained from the Cleveland Blood Center on a contractual basis) by Ficoll-Hypaque Plus density centrifugation. CD34^+^ hematopoietic stem and progenitor cells (HSPCs) were purified by positive selection using the Midi-magnetic–activated cell sorting LS columns (130-042-401, Miltenyi Biotec). CD34^+^ cells were cultured in Iscove modified Dulbecco medium (IMDM) containing 20% BIT 9500 medium (Stem Cell Technologies) supplemented with SCF (100 ng/mL), Fms-like tyrosine kinase 3 (FLT-3; 10 ng/mL), IL-6 (20 ng/mL), and thrombopoietin (TPO; 100 ng/mL; these cytokines were purchased from PeproTech).

### Generation of retroviruses and infection of primary hematopoietic CD34^+^ cells.

Retrovirus encoding WT *DNMT3A* or *DNMT3AR882H* mutant or empty vector were produced by transfection of 293T cells. After 24 hours of culture, CD34^+^ cells were infected with high-titer virus in the presence of 8 μg/mL polybrene (Sigma-Aldrich). GFP-sorted CD34^+^ cells were subjected to colony assay as described below. Sorted GFP^+^ cells were subjected to total RNA isolation using the RNeasy mini kit (QIAGEN) and submitted for RNA-Seq.

### Colony-forming assay.

HSPCs were isolated using Mouse Hematopoietic Progenitor Cell Isolation Kit following manufacturer’s instructions (BioLegend). Unfractionated BM cells collected from *Dnmt3a^–/–^* mice were subjected to HSPC enrichment. HSPCs were plated in 6-well plates (Corning) containing 1 mL of complete methylcellulose medium in the presence and absence of the PI3K αβ inhibitor (M3434, Stem Cell Technologies; containing 15% FBS, 50 ng/mL rmSCF, 10 ng/mL rmIL-3, 10 ng/mL rhIL-6, 3 U/mL rhEPO). This medium was supplemented with 10 ng/mL Flt-3 (R&D Systems) and 10 ng/mL TPO (R&D Systems). Cultures were maintained at 37°C in humidified chambers containing 6% CO_2_. Colony formation was scored after 8–10 days of culture. Total number of colonies and total number of cells from each well was quantitated. Cells from the primary colony were replated in 6-well plates and cultured under the same conditions as in the first round but without PI3K αβ inhibitor for 8–10 days. Subsequently, colony numbers were assessed, and single-cell suspensions were subjected to flow cytometry using an anti–Mac1 antibody (101206, BioLegend) to assess differentiation of myeloid cells.

### Cell migration assay with enriched CD45^+^ cells from Dnmt3a^–/–^ mouse liver tissues.

Liver tissues were harvested from hepatomegaly presented malignant *Dnmt3a^–/–^* mice or similarly aged *Dnmt3a^+/+^* mice and homogenized by shearing followed by CD45 enrichment using CD45 negative selection kit (MagniSort Mouse CD45 depletion kit, Invitrogen). Magnetic bead–bound cells were assessed for percent CD45 enrichment using flow cytometry. Cell invasion and migration assay was performed as previously described (58). Briefly, transwell filters (6.5 μM pore filter; Costar) were placed in the lower chamber containing 500 μL of complete medium with or without SCF (100 ng/mL). Enriched CD45 cells (2.5 × 10^5^) from *Dnmt3a^–/–^* liver tissues were resuspended in 500 μL RPMI media in the presence or absence of 100 nM PI3K αβ inhibitor (Bay1082439) or 0.5 μM RAC1/3 inhibitor (EHop-016; Selleckchem) or 200 nM RAC/CDC42 inhibitor (MBQ-167; MCE) and allowed to migrate toward the bottom of the top chamber. After 20 hours of incubation at 37°C, nonmigrated cells in the upper chamber were removed with a cotton swab. The migrated cells that attached to the bottom surface of the membrane were stained with 0.1% crystal violet dissolved in 0.1M borate (pH 9.0) and 2% ethanol for 5 minutes at room temperature. The number of migrated cells per membrane was counted in 10 random fields with an inverted microscope using 20× objective lens.

### Cytokines, antibodies, and reagents.

Recombinant murine IL-3 and SCF were purchased from Pepro Tech. Antibodies to conduct flow cytometry analysis: c-KIT (catalog 105814), B220 (catalog 103208), CD3 (catalog 100320), Sca-1 (catalog 122512), CD16/32 (catalog 101328), CD34 (catalog 562608), CD127 (catalog 135014), CD19 (catalog 302217), CD71 (catalog 113812), CD11b (catalog 101206), Ly6C/Ly6G (catalog 101424), CD48 (catalog 105432), CD150 (catalog 115921), mouse CD45 (catalog 103112), mouse CD45.1 (catalog 110736), mouse CD45.2 (catalog 109832), human CD45 (catalog 304008), and human Ter119 (catalog 116208) were purchased from BioLegend. CD4 (catalog 553730), CD8 (catalog 553033) antibodies purchased from BD Biosciences. Anti-DNMT3a (catalog NB120-13888) antibody was purchased from Nouvus Biologicals. Anti–phospho-AKT Ser473 (catalog 4060), and anti-AKT (9272) from Cell signaling Technology (Beverly, MA), and anti–β-actin antibody from MilliporeSigma. IMDM was purchased from Invitrogen. [^3^H] Thymidine was purchased from PerkinElmer. PI3K αδ inhibitor GDC-0941 was purchased from Selleck Chemicals, and PI3K αβ inhibitor Bay1082439 was purchased from MedKoo Biosciences. Flow cytometry for Intracellular phosphor-AKT (ser 473) levels in stem cells was performed manufacturer protocol (BD Phosflow). Phospho-Akt (Ser473) (D9E) rabbit monoclonal antibody (Alexa Fluor 647 Conjugate) was purchased from Cell Signaling Technology (catalog 4075).

### Western blotting.

Equal amount of protein extracts was separated on 4%–20% SDS-polyacrylamide gels. After electrophoresis, the proteins were transferred onto nitrocellulose membranes, and nonspecific binding was blocked with 5% nonfat dry milk in Tris-buffered saline containing 0.1% Tween-20 (TBS-T). Membranes were then probed with various antibodies overnight at 4°C on a rocker. After incubation, membranes were washed with TBS-T and incubated with appropriate horse radish peroxidase–conjugated (HRP-conjugated) secondary antibodies for 1 hour at room temperature. After washing the membranes with TBS-T, the proteins on the membranes were detected using Super Signal West Dura Luminol/Enhancer solution (Thermo Fisher Scientific) and by exposing the membranes to x-ray film.

### Histopathology.

Spleen, liver, and thymus samples were fixed in 10% neutral buffered formalin and paraffin embedded. Thin sections (5 μm) were cut on a microtome and stained with H&E using standard protocols. All histology slides were hand read by a pathologist. All images obtained were scanned using Aperio Whole Slide Digital Imaging Platform.

### Genome-wide transcript analysis using RNA deep sequencing.

Twelve- to 16-week-old *Dnmt3a*^fl/fl^:*Mx1-*Cre^–^ or *Dnmt3a*^fl/fl:^*Mx1-*Cre^+^ mice were administered with poly I:C (i.p. injected with 150 μg poly I:C 3 times with 48-hour time intervals), and 4 weeks after poly I:C administration BM cells were harvested, BMNCs were separated using Ficoll-Paque density gradient centrifugation (400*g* for 30 minutes at room temperature). Cells were cultured in DMEM supplemented with SCF (50 ng/mL) and IL-3 (10 ng/mL) for 48 hours and later with IL-3 (10 ng/mL) for 1 week. RNA was isolated from cultured BMNCs derived from 3 WT mice and 3 *Dnmt3a^–/–^* mice and subjected to sequencing for genome-wide transcript analysis (GSE208435) using the llumina platform at BGI sequencing services (BGI Americas). In addition, we also performed genome-wide transcript sequencing analysis on whole BM cells derived from vehicle and PI3K αβ inhibitor–treated mice using the llumina platform at CMG sequencing core at Indiana University (GSE208437). We collected the raw sequence reads (paired-end reads) and ensured the quality of the reads using FastQC (default parameter). The raw sequences were checked for adaptor content and for high content of any unknown bases (N) reads to ultimately get the clean reads. The data are specific to BM cells and have paired-end fragments with an average of 101 bp read length. Hierarchical Indexing for Spliced Alignment of Transcripts (HISAT) was set at default and used for aligning the quality filtered RNA sequence reads against the whole mouse reference genome mm10. Sequence Alignment Map (SAM) files and the output files from HISAT were processed using SAMtools (version 0.1.19) and subsequently used for the quantification of transcript expression levels. In order to identify and quantify transcripts from RNA-Seq reads, StringTie (Version 1.2.1) was used with default parameters to quantitate the transcripts of each genomic locus, considering all possible multiple splice events. Fragments per kilobase of transcript per million mapped reads (FPKM) values for each gene were considered for further downstream analysis. Empirical analysis of digital gene expression data in R (EdgeR) package was used for differential gene expression analysis of RNA-Seq expression profiles. StringTie output of genes and their respective FPKM values were converted to data matrix, and thereby, differentially expressed genes were identified. Only genes that were differentially expressed with a FDR < 0.01 were considered for further functional enrichment and GSEA.

### GSEA.

GSEA was performed using the default parameters, except Enrichment Statistic Classic instead of weighted, using GSEA desktop application (version 2.2.2). All of the genes obtained from StringTie analysis with log-fold changes and *P* values from EdgeR were included to construct a rank order by using a scoring metric. Eventually, we examined the upregulated or downregulated genes involved in biological processes from the KEGG database.

### Ingenuity pathway analysis of genes.

Genes enriched for pathways were analyzed using the ingenuity pathway analysis tool following instructions from the provider (Qiazen). Differentially expressed genes with *P* values less than 0.05 and FPKM values greater than 15 were included in the analysis to understand the significance of expressed transcripts in respective biological processes. Differentially expressed genes with log fold change greater than 1.5 (for upregulated processes) and less than –1.5 (for downregulated processes) were considered.

### qPCR assay.

BM cells from mice bearing *Dnmt3a^–/–^* cells were treated with vehicle or PI3K αβ inhibitor as in Figure 2A and flow sorted for CD45.2^+^ *Dnmt3a*-null cells. Sorted cells were subjected to qPCR analysis using the following oligos. *Cdc42bpb* (F: 5′-CCG AGA CAT TCC GTG CAT ATT-3′, R: 5′-ATC CCT ACC CAC TTC CTC TTT-3′), *Rho-GEF5* (F: 5′-CCT GTT CCT GTG TTG TCT TCT-3′, R: 5′-CTT GGC GAT CTC AGG AAT GT-3′), *Vcam1* (F:5′-GCA CTC TAC TGC GCA TCT T-3′, R: 5′-CAC CAG ACT GTA CGA TCC TTT C-3′), *Farp2* (F: 5′-GCA AGG AAG CTG GAA ATG TAT G-3′, R: 5′-CAG GAC ACC CAT GTG AGA AA-3′), *Limk1* (F: 5′-ATC CAG GTA GCA TCA GGT ATT G-3′, R: 5′-TGA GAT GGA CAC TAA GCA AGA G-3′), *Mpeg1* (F: 5′-GAA AGA GAG CAA CCT GGA GAT G-3′, R: 5′-GGG CGA GTT CTG TGT TGA TAG-3′), *Arpin* (F: 5′-ATC TCT GTC TTA GGG CTC TTA CT-3′, R: 5′-CTC CTG CTC TCA CTG CTT TAC-3′), *Cfl2* (F: 5′-GTG TGC GCT CTC TGC TAT TA-3′, Coflin2 R: 5′-TGC CAT CTG TGG ATG ACT ATG-3′), *Arp2* (F: 5′-CAG TCA CAG TGG AAT CCC TAA G-3′, R: 5′-TCC TAT GCA CAG CAG GTA ATG-3′). Relative gene expression to *GAPDH* expression levels were plotted. Oligos were purchased from IDT.

### Statistics.

Statistical analyses were performed using GraphPad Prism software. Unpaired *t* test (2-tailed) was used for statistical comparison between 2 groups. Ordinary 1- or 2-way ANOVA with Tukey’s multiple comparison or Sidak’s multiple-comparison test were performed for statistical comparisons.

### Study approval.

All studies were approved by Indiana University Laboratory Animal Resource Center. All animals were maintained in a pathogen-free facility at Indiana University School of Medicine. All animal procedures were conducted in accordance with the *Guide for the Care and Use of Laboratory Animals* (National Academies Press, 2011) and were approved by the IACUC at Indiana University School of Medicine.

## Author contributions

LRP and R Kapur conceptualized the study, designed the experiment, analyzed the data, and wrote the manuscript. BR, KP, FP, MS, BS, GS, UPD, R Kanumuri, AC, and SKP assisted with experiments and provided scientific input. SP conceptualized the study.

## Supplementary Material

Supplemental data

## Version 1 03/28/2023

In-Press Preview

## Version 2 05/08/2023

Electronic publication
