# Supplementary material for: Loss of Dnmt3a impairs hematopoietic homeostasis and myeloid cell skewing via the PI3Kinase pathway
Source: JCI Insight. 2023 May 8;8(9):e163864. doi: 10.1172/jci.insight.163864 (PMC10243813; doi:10.1172/jci.insight.163864)
Supplement: Supplemental data [file jciinsight-8-163864-s218.pdf]

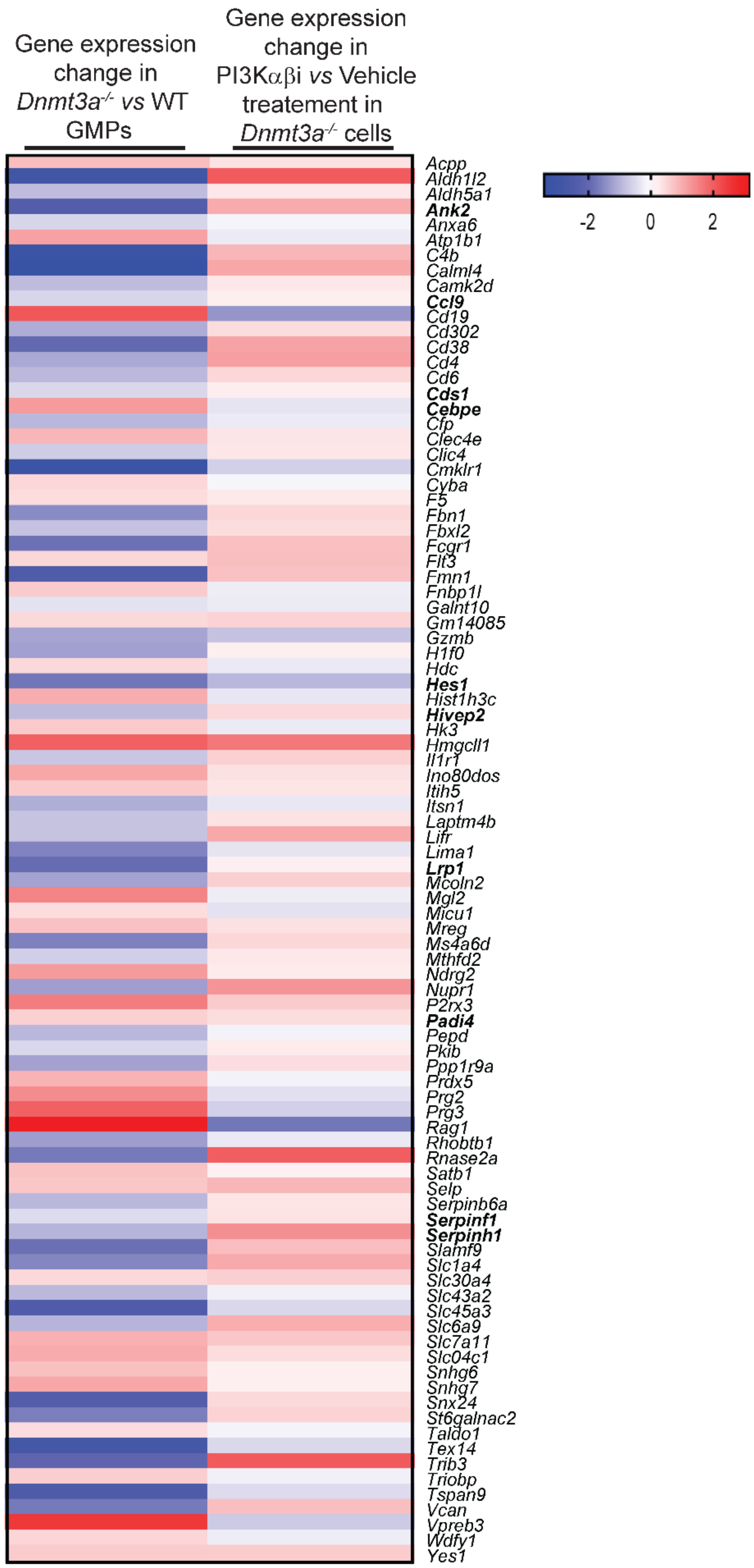

Supplementary Figure 1 (Figure 8E related): Comparative analysis of GMP progenitor gene expression data involving *Dnmt3a*$^{-/-}$ and wildtype mice (13) with the BM derived gene expression data in the current study from vehicle and PI3Kinase αβ treated mice bearing *Dnmt3a* deleted malignant cells. 93 genes were found to be differentially regulated.

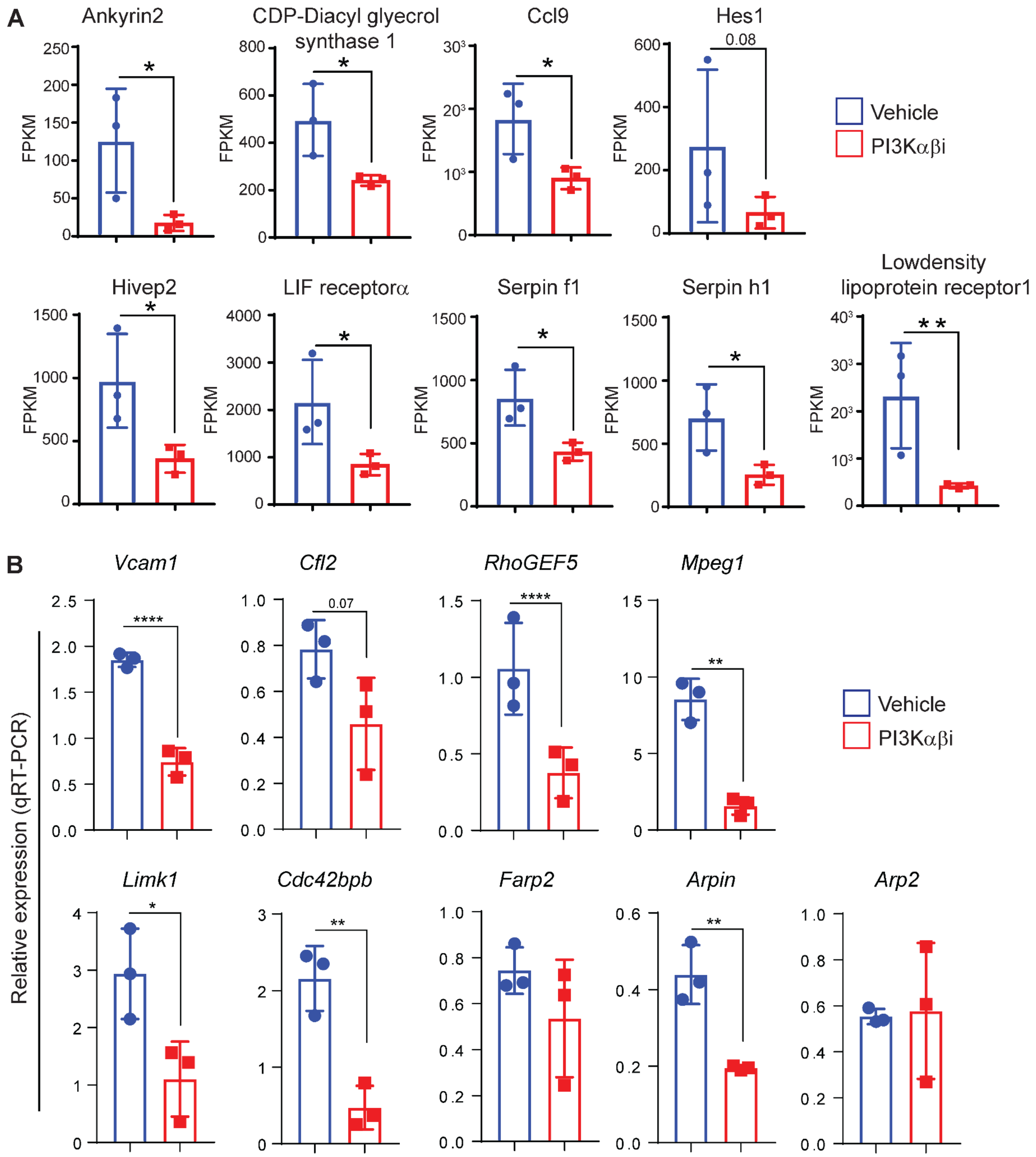

Supplementary Figure 2 (A). (Figure 8F related): PI3K inhibition reduces gene expression of genes in cell motility, migration, and tumor invasiveness. Gene expression levels of indicated genes in vehicle and PI3Kαβ inhibitor treatment conditions are shown. n=3, ± SEM, *p=0.05, **p=0.005. (B). (Figure 9A related): PI3K inhibition reduces RAC1/CDC42 pathway related gene expression in *Dnmt3a*$^{-/-}$ cells. The expression of indicated genes was validated by qRT-PCR on sorted CD45.2+ cells (*Dnmt3a*$^{-/-}$) from vehicle or PI3Kαβ inhibitor treated F1 mice as in Figure 2A. n=3, ± SEM, *p=0.05, **p=0.005, ****p=0.00005. Unpaired t-test (two-tailed) performed (A-B).

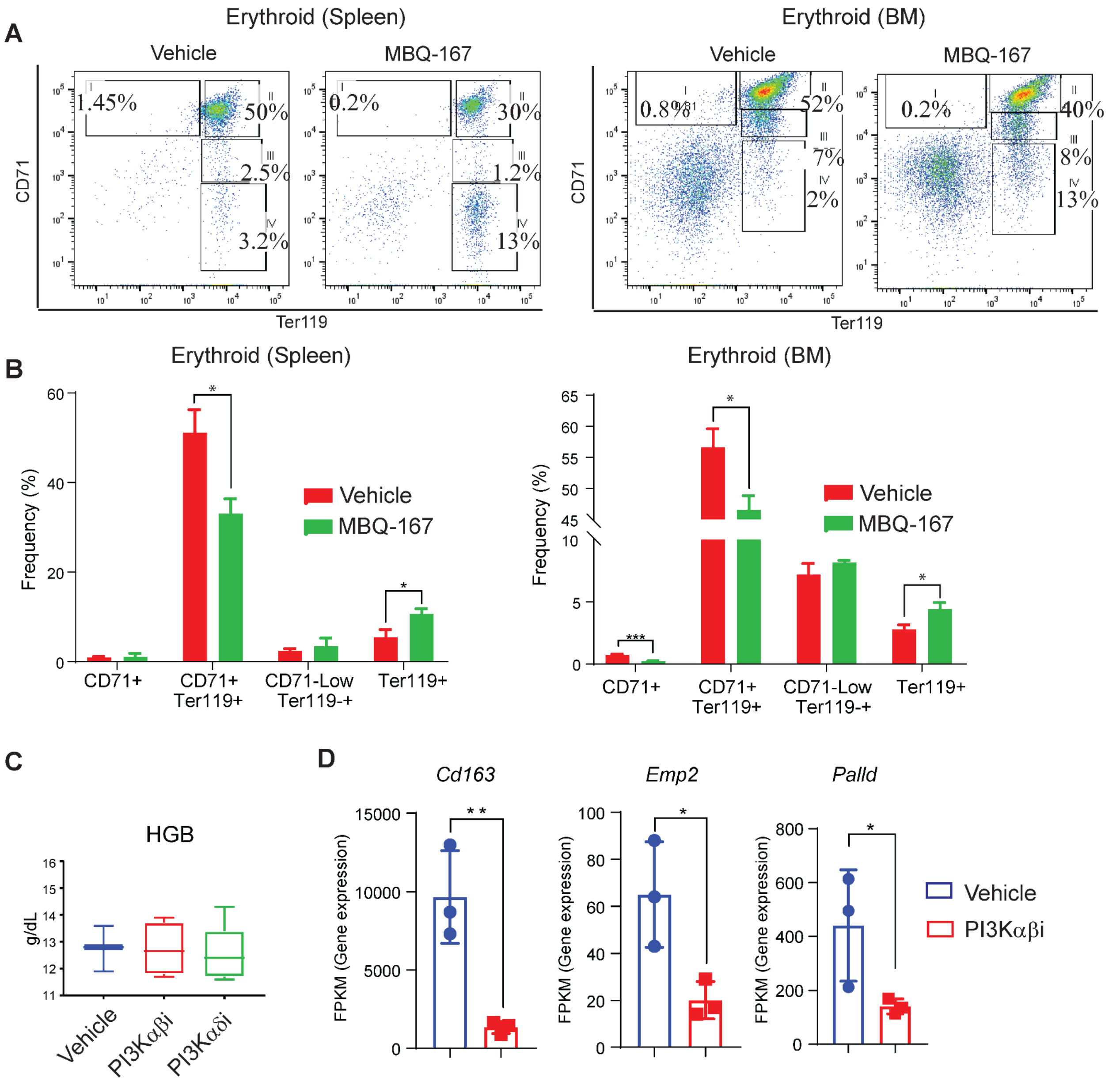

Supplementary Figure 3. Rac/CDC42 pathway inhibition improves erythroid cell maturation in mice bearing *Dnmt3a*$^{-/-}$ malignant cells. (A) Flow cytometry analysis was performed on spleen and BM cells from mice transplanted with *Dnmt3a*$^{-/-}$ cells and treated with the vehicle or MBQ-167. Shown are representative dot plots with CD71+, Ter119+ cells. (B) Quantitative data showing increased presence of mature erythroid cells in BM and spleen of RAC/CDC42 pathway inhibitor treated mice compared to vehicle. n=5-6, ± SEM, 2way ANOVA *p=0.05. (C) Hemoglobin quantitation plot related to PB counts from mice described in (Figure 2B & C) before they were sacrificed. n= 3-5, The boxes shown with lower and upper quartiles separated by the median (horizontal line) and the whiskers extend to the minimum and maximum values. (D) Quantitative gene expression data showing the expression of *Cd163*, *Emp2* and *Palld* genes from the indicated groups. n=3, *p=0.05, ± SEM, Unpaired t-test (two-tailed) **p=0.005.
